# How Free-Viewing Eye Movements Can Be Used to Detect the Presence of Visual Field Defects in Glaucoma Patients

**DOI:** 10.3389/fmed.2021.689910

**Published:** 2021-10-21

**Authors:** Birte Gestefeld, Jan-Bernard Marsman, Frans W. Cornelissen

**Affiliations:** ^1^Laboratory for Experimental Ophthalmology, University of Groningen, University Medical Center Groningen, Groningen, Netherlands; ^2^Department of Neuroscience, Research School of Behavioral and Cognitive Neurosciences Neuro-Imaging Center, University Medical Center Groningen, Groningen, Netherlands

**Keywords:** visual field defects, glaucoma, eye tracking, free-viewing, viewing priority

## Abstract

**Purpose:** There is a need for more intuitive perimetric screening methods, which can also be performed by elderly people and children currently unable to perform standard automated perimetry (SAP). Ideally, these methods should also be easier to administer, such that they may be used outside of a regular clinical environment. We evaluated the suitability of various methodological and analytical approaches for detecting and localizing VFD in glaucoma patients, based on eye movement recordings.

**Methods:** The present study consisted of two experiments. In experiment 1, we collected data from 20 glaucoma patients and 20 age-matched controls, who monocularly viewed 28 1-min video clips while their eyes were being tracked. In experiment 2, we re-analyzed a published dataset, that contained data of 44 glaucoma patients and 32 age-matched controls who had binocularly viewed three longer-duration (3, 5, and 7 min) video clips. For both experiments, we first examined if the two groups differed in the basic properties of their fixations and saccades. In addition, we computed the viewing priority (VP) of each participant. Following a previously reported approach, for each participant, we mapped their fixation locations and used kernel Principal Component Analysis (kPCA) to distinguish patients from controls. Finally, we attempted to reconstruct the location of a patient's VFD by mapping the relative fixation frequency and the VP across their visual field.

**Results:** We found direction dependent saccade amplitudes in glaucoma patients that often differed from those of the controls. Moreover, the kPCA indicated that the fixation maps of the two groups separated into two clusters based on the first two principal components. On average, glaucoma patients had a significantly lower VP than the controls, with this decrease depending on the specific video viewed.

**Conclusions:** It is possible to detect the presence of VFD in glaucoma patients based on their gaze behavior made during video viewing. While this corroborates earlier conclusions, we show that it requires participants to view the videos monocularly. Nevertheless, we could not reconstruct the VFD with any of the evaluated methods, possibly due to compensatory eye movements made by the glaucoma patients.

## Introduction

Screening for visual field defects (VFD) as well as monitoring their progression is critical in the management of many ophthalmic diseases, such as glaucoma. The risk for developing a VFD increases with age. It is crucial to detect its presence early on, especially in glaucoma patients who, eventually, will become blind if disease progression is not halted through treatment. The current gold standard method for detecting visual field defects (VFD) is standard automated perimetry (SAP). It requires the tested person to fixate their gaze on a single point and to press a button every time that they perceive a stimulus in the periphery of their visual field. This means that they have to maintain a high level of attention over a prolonged period of time. It is also required that the participant correctly understands the task. As a consequence, SAP is rather difficult to perform for children under the age of seven years, many elderly people, and people with attentional problems (1–3). This is problematic, as non-compliance will increase test duration and can negatively impact the precision of the measurement (4). Therefore, there is a need for screening tests which are easier to perform and more engaging than the current ones.

### Exploiting Naturally Occurring Eye Movements to Monitor the Occurrence of VFD

One of the most difficult aspects of SAP is that the patient needs to fixate a single point while attending to stimuli appearing in their visual periphery. Rather, one's natural tendency would be to make an eye movement to such suddenly appearing stimuli. In our view, rather than asking the patient to suppress such reflexive tendencies, one should try to exploit these tendencies when screening for the presence of a VFD. Previous studies have attempted to simplify the gold standard perimetric test by instructing participants to make saccades toward the target stimuli presented in the periphery, instead of pressing a button while maintaining fixation. Previous studies found that the saccadic reaction time of glaucoma patients is significantly lower than those of normal-sighted controls and concluded that this feature could be exploited in a new type of perimetric screening test (5, 6). In our current study, our specific goal was to avoid giving our participants a task on which they had to focus, and present stimulus material that is naturally engaging for most people and that would evoke spontaneous viewing behavior. This is why we chose to show short video clips and simply asked our participants to just view and enjoy them, while we recorded their eye movements. If we could find differences in the free-viewing eye movement behavior of glaucoma patients and normal-sighted controls using this approach, this could result in a very intuitive way of screening for the presence of a VFD. Such an approach could potentially be more suitable for use in people that have trouble with performing SAP. However, such variegated target groups are less suitable for evaluating a new approach, which is why in the present study we focus on adult glaucoma patients.

### VFD Influence Eye Movements

Previous research has shown that patients with VFD differ from normal-sighted controls in various eye movement features. However, these differences are often subtle and the results of studies could differ depending on their experimental paradigm. Observers with simulated VFD showed longer fixation durations during visual search (7). Moreover, Smith et al. (8) found that glaucoma patients made fewer saccades during visual search than normal-sighted controls. In contrast, Wiecek et al. (9) found no differences in participants with actual VFD, compared to controls, in search and fixation duration, saccade amplitude or the number of saccades made. These differences between visual search studies may be attributed to differences in experimental design and whether the VFD was real or simulated. While Cornelissen et al. (7) used computer generated search displays containing Landolt-C's and simulated the VFD, the other two studies used photographs of natural scenes, and included participants with actual VFD. However, the two latter studies differed in that they either recorded binocular (8) or monocular (9) viewing behavior. These examples suggest that differences in experimental design may strongly affect the outcome of studies on the influence of VFD on eye movements.

### Cross Validating Previous Methods of Analysis

Previous studies have shown that it is possible to detect the presence of either real or simulated VFD based on free viewing eye movements (10–12). Moreover, simulated VFD could even be reconstructed based on an analysis of these free viewing eye movements (12). However, there have only been few studies on this topic and the methods that were used have not been cross-validated on different data sets. In addition, it is critical to verify whether results that have been achieved with simulated VFD can be replicated in participants with real VFD. It is known that patients experience their loss of sensitivity in the visual field differently from how VFD are simulated (13). Previously, we simulated the VFD by masking parts of the visual field with a gray-level bitmap, resulting in a VFD which was noticeable, instantaneous and blocked parts of the visual field completely (12). A real glaucomatous VFD does not have any of these attributes. Often, glaucoma patients are not even aware of their VFD due to their brain filling-in the missing information (14). The consequence of filling-in on viewing behavior is unknown. Therefore, while it is obvious that simulated and real VFD differ in many ways, the consequences thereof, for our ability to use eye movements to detect VFD, is not. Consequently, it is critical to evaluate whether analysis methods are also suitable for detecting VFD in actual patients.

The present study consists of two experiments with slightly different paradigms. For our first experiment, we collected eye movement data from 20 glaucoma patients and 20 age-matched controls, who watched 28 video clips of 1-min length with one eye. In the second experiment, we reanalyzed a data set (15) in which 44 glaucoma patients and 31 controls watched three different video clips of varying length with both eyes simultaneously. All glaucoma patients who participated in these two experiments had asymmetrical visual field loss, which meant that the state of their visual field changed depending on whether they viewed the stimulus with one or with both eyes.

We hypothesized that the free-viewing eye movements of the glaucoma patients, made while viewing video clips, would differ from those of normal-sighted controls. More specifically, we tested whether we could detect and localize the VFD. Since our comparison will be to gold standard SAP, which is performed monocularly, in experiment 1 we choose to record viewing behavior monocularly. This will allow us to compare the VFD reconstruction based on viewing behavior to visual field sensitivity as determined by SAP. In contrast, the eye movement data of experiment 2 was recorded during binocular viewing. A comparison over studies may allow us to answer if using one or two eyes to view videos leads to different viewing behavior in patients and which approach is most suitable for screening for VFD. We performed nearly identical analyses on both data sets. This way, we could determine which analyses, experimental conditions and video materials best separate patients and controls.

First, we compared the group medians of different basic eye movement features. We also compared the viewing priority (VP) of the two groups (16). VP can be used to express the similarity in the viewing behavior of an individual to that of a peer-group of observers watching the same movie, while taking into account any inherent biases (e.g., center bias) in viewing behavior that may be present (e.g., induced by the use of a specific set-up, such as a screen). The VP algorithm takes the viewing behavior of a large group of observers, both made during a specific movie and during a number of other movies, as the starting point for its comparison. The VP of a given fixation is calculated using fuzzy c-means clustering. First, the distance to a set of reference fixations is measured. Reference fixations are fixations made by other observers during the same time interval, while watching the same video clip. This means that we compare the locations of fixations of one participant to those of other participants, while they are viewing the same content. In addition, the distance of the reference fixations to a set of random fixations are calculated. Random fixations are fixations made by other observers during the same time interval, while watching a different video clip. In summary, VP measures the similarity of the viewing behavior of a selected observer to and a reference group, while taking into account content independent biases.

In addition, we cross validated and replicated another method of analysis which had previously been used to separate glaucoma patients and controls, namely kernel principal component analysis (kPCA) in combination with naive Bayes classification (10). In kPCA, the original data, in the case of this study the proportion of saccade end points in a grid across the visual field, is transformed into a higher dimensional space, where it can be linearly separated. For the purpose of this study, it would be ideal if the patients could be separated from the controls in this new space.

In addition to separating the two groups, we attempted to reconstruct the location of the VFD using two different methods. We computed the distribution of the VP and the distribution of fixation frequency over the visual field aiming at identifying damaged regions in the visual field based on a reduced VP or a deviating frequency of fixation from the control group.

The underlying assumption of this analysis is that patients with VFD would systematically miss salient information in damaged parts of their visual field, due to a reduction in the bottom-up information. As such, in the absence of any compensatory strategies, they would be expected to make fewer saccades toward the damaged part of their VF. Using a top-down strategy, patients may try to compensate for their VFD by frequently directing their gaze toward the damaged parts of the visual field. If successful, the distribution of fixations across the visual field would not differ between patients and controls. In fact, a patient may even fixate the damaged parts of the visual field more frequently than the controls. However, in such a case, the viewing priority in the damaged areas of the visual field of the patients would be lower, because such compensatory eye movements would not necessarily be directed at conspicuous events or parts in the scene.

Next, the methods and results for each of the two experiments will be described in separate sections. In the discussion, we will compare and discuss the results of the two experiments.

**Experiment 1:** Monocular eye movements under free-viewing conditions of glaucoma patients compared to those of normal sighted observers.
**Experiment 2:** Binocular eye movements under free-viewing conditions of glaucoma patients compared to those of normal sighted observers.

## Experiment 1: Monocular Eye Movements Under Free-Viewing Conditions of Glaucoma Patients Compared to Those of Normal Sighted Observers

### Methods

#### Summary

In this experiment, we collected eye movement data of glaucoma patients and age matched controls, who each viewed a large number of short video clips. We occluded one eye, with the aim of eliminating any putative compensation of the VFD between the two eyes in the glaucoma patients, and obtaining results under similar conditions as in SAP.

Showing short video clips also allowed us to analyze the eye movement features for each clip separately and investigate the influence of video content on eye movements.

#### Participants

We collected data from 31 glaucoma patients and 32 controls. The patients with VFD had a mean age of 64 years (range: 40–81 years) and the controls had a mean age of 60 years (range: 35–83). Details can be found in Table 1. All participants had normal or corrected to normal visual acuity. We had to exclude the data of 11 glaucoma patients and 12 controls, due to an inability of the eye tracker to continuously measure the gaze position, due to a loss of the pupil or the corneal reflection. This was the case for participants (Glaucoma patients and controls) who wore multifocal glasses, participants with drooping eyelids or participants who had artificial lenses. As the eye tracker could not be used with multifocal glasses, we replaced them with a pair of trial lenses from the ophthalmology clinic, with the appropriate correction. However, this did not always enable the eye tracker to acquire data continuously.

**Table 1:** Characteristics of the participants.

**Participant characteristics**	**Controls**	**Patients**
Age range (in years)	35–83	40–81
Mean age (+SD)	60 (10.39)	64 (12.19)
Gender (in percentage)	male = 55%	male = 55%

The ethics committee of the UMCG approved the study protocol. All participants provided written informed consent. The study followed the tenets of the Declaration of Helsinki.

#### Procedure

All participants watched 28 different video clips of 1-min length with one eye. The clips were taken from different types of videos, such as motion pictures, nature documentaries or comic films. The list of videos from which they were taken is provided in the Supplementary Table 1.

At the beginning of each session, we performed a standard 9-point calibration choose with which eye the participant would perform the experiment at random. Next, we performed a standard 9-point calibration using the built-in routines of the tracker and tested if we could obtain an accurate calibration. In case we could not obtain an accurate calibration, we also obtained the calibration accuracy for the other eye and performed the experiment with the most accurately calibrated eye. Details on the visual field of the glaucoma patients and on which eye was tracked in the experiment can be found in Table 2. Participants were seated at 60 cm distance from the screen and were asked to place their head in a chin rest. They were asked to view the video clips as they would normally. We gave the participants the opportunity to take a break after each video clip.

**Table 2:** This table shows the age, gender, with which eye they performed the experiment, the MD value of the tested and the covered eye and the IVF score of each glaucoma patient.

**Patient ID**	**Age**	**Gender**	**Eye tested**	**MD (tested/covered eye)**	**IVF score**
P003	70	Male	Left	−6.65 / −26.26	14
P004	73	Male	Right	−19.84 / −10.28	21
P008	64	Male	Left	−1.42 / −15.31	0
P009	66	Female	Right	−4.86 / −9.85	4
P010	69	Female	Right	−17.24 / −6.96	10
P013	41	Male	Right	−28.98 / −28.96	88
P014	72	Female	Left	−32.76 / −23.74	79
P016	69	Male	Left	−16.45 / −24.48	77
P021	78	Male	Right	−6 / 0.61	0
P022	65	Female	Left	−3.34 / −1.56	0
P023	47	Female	Left	−5.24 / −5.29	0
P025	78	Male	Left	−5.81 / −20.23	12
P026	81	Male	Right	−24.9 / −2.78	7
P027	67	Male	Left	−23.58 / −16.76	37
P028	60	Male	Left	−24.49 / −24.07	72
P029	64	Male	Right	Only FDT available	n.a.
P030	77	Male	Left	−17.99 / −15.75	65
P031	63	Female	Right	−8.68 / −0.93	3
P032	68	Female	Right	−6.21 / −5.82	2

The clinical data of the patients (visual fields and visual acuity) was obtained from their medical record at the UMCG. For the control group, either before or after they viewed the video clips, we measured eye pressure, visual acuity, and used a frequency doubling technology (FDT) perimeter-based screening and an OCT image to rule out the presence of glaucoma and/or a VFD. In FDT, the participant has to fixate in the middle of the stimulus display, while flickering achromatic sinusoidal grating of low spatial frequency is presented. The participant has to press a button when they see a stimulus. A deviant FDT score is indicative of putative retinal ganglion cell damage associated with glaucoma (17). In addition, we performed a Montreal cognitive assessment test (18) with all participants to rule out the presence of cognitive deficits.

#### Stimulus Presentation and Eye Tracking

We presented the video clips full-screen on a 50 cm by 35 cm (1,920 × 1,080 pixel) display (BenQ Zowie xl2540). Participants viewed the screen from a distance of 60 cm, such that it covered a visual field of 45.2 × 32.5 deg (of visual angle). One eye of the participants was covered with a standard ophthalmic eye patch. Monocular eye movements were recorded with an Eyelink 1000 and an Eyelink duo eye tracker (SR Research) at 1,000 Hz. The host PC was connected to a laptop running MATLAB (Version 2017b, MathWorks, Natick, MA) with the Psychtoolbox (19, 20) and the Eyelinktoolbox (21) *via* Ethernet. All video clips were presented in the same order for each participant. The procedure was controlled by a custom Matlab script.

#### Selection of the Video Clips

We used a subset of the movies shown in Gestefeld et al. (12). The movies were selected because, based on data recorded in the experiment of Gestefeld et al. (12), they resulted in different viewing behavior in observers with and without various simulated VFD.

The video clips we used for this study were selected based on the classification performance of a k-nearest neighbor (kNN) classifier distinguishing between four different classes (the three different simulated glaucoma archetypes and the control conditions) using data from each video clip. We then selected the video clips which showed a classification performance of ≥50%. This resulted in 28 1-min video clips.

#### Data Analysis

##### Fixations and Saccades in Visual Field Coordinates

The Eyelink 1000 processes the raw data using its built-in algorithms to define fixations and saccades. Saccades were defined using a velocity threshold of 30 deg/s. and an acceleration threshold 8,000 deg/s^2^. All other eye movement data was classified as a fixation. We used this pre-processed data for further analysis. The fixation location and saccade start and end points were provided as x-y coordinates on the screen, where the origin of the screen was the top left corner.

As the location of the visual field changes its location with every eye movement with respect to the scene, we define its origin at the starting point of each saccade. When analyzing fixations, we define the center of the visual field at the location of a fixation at a certain time point. The position of the next fixation was then determined with respect to this fixation of which the location was the origin of the coordinate system. In terms of these visual field coordinates, eye movements to the left result in a negative coordinate along the x-axis and downward eye movements result in a negative coordinate along the y-axis.

##### Basic Eye Movement Features

We first computed the mean fixation duration, the number of fixations, the mean saccade amplitude and the mean saccade velocity over all video clips for each subject and compared the two groups using a Wilcoxon-Mann-Whitney signed-rank test. A *p*-value of *p* < 0.05 was regarded as significant. As we compared three basic eye movement and, in a later analysis step, the VP of the two groups we corrected for four measurements. After Bonferroni correction for multiple comparisons a *p*-value of *p* < 0.013 was regarded as significant.

#### Comparison of Directional Saccade Amplitudes of Glaucoma Patients and Controls

Then we tested if the directional saccade amplitudes of individual glaucoma patients differed from those of the control group. We first estimated a “normal” range of median and maximum saccade amplitudes by computing them for all participants in the control group. We then determined whether the median and maximum saccade amplitude of each patient with VFD stayed within this “normal” range.

More precisely, we computed the median and maximum saccade amplitudes of each participant in 18 bins of 20 deg of visual angle, spanning 360 deg. For each of the glaucoma patients we computed the rank of the median and maximum saccade amplitude relative to those of the controls in the same directional bin. The rank order was normalized by dividing it by the number of controls, so that it ranged between 0 and 1.We then plotted the rank of the saccade amplitude in each direction and marked the upper and lower 2.5% of the normalized ranks with a dashed line (see **Figure 2**).

To see if patients with similar types of VFD, in the eye that they used to watch the video clips, showed similar patterns in directional saccade amplitudes, we split our patients into six different groups according to the VFD in the tracked eye: peripheral VFD (scotoma outside the central 10 deg), nasal arc, large peripheral VFD (tunnel vision), VFD affecting the periphery and the central 10 deg of the visual field, almost complete blindness and intact visual field. We did not perform any statistical inference tests, as we only had small groups of participants with a similar type of VFD.

#### Viewing Priority

We calculated the viewing priority (VP) with the aim to determine whether the fixation locations of (individual) glaucoma patients differ systematically from those of the normal-sighted controls. We expect to find an overall lower VP in glaucoma patients, if they frequently fail to direct their gaze toward parts of the visual scene, which get fixated by the control group. In addition, we could use the VP to localize damaged parts of the visual field, if the VP of fixations after eye movements toward the damaged parts of the visual field was systematically decreased.

We extracted the fixations of each participant from the pre-processed data and computed the VP for each of them (12, 16). To compute the VP of a fixation by one of our participants, we need a “reference set” of fixations and a “random set” of fixations, both consisting of fixations made by other participants. The more densely the reference fixations are clustered in the same region, compared to the random fixations, and the closer the fixation of interest is located to the reference fixations, the higher is the VP.

For both reference and random data set we used fixations made by the control group, because we know that the viewing behavior of normal-sighted observers during movie viewing is very consistent, with the fixations of most observers clustering in the same areas of a scene. Also, the aim of this study is to identify deviances of the glaucoma patients viewing behavior from normal viewing behavior. VP provides a very straight-forward way to identify them.

We averaged the VP value from all fixations of each trial for each participant and used a Wilcoxon-Mann-Whitney test to compare the ranks of the two groups. After Bonferroni correction, a *p*-value <0.002 was regarded as significant. In addition, we computed the Pearson's correlation coefficient of the VP and the severity of the VFD. More precisely, we correlated the VP with the mean deviation (MD) of the luminance sensitivity (in dB) from normal as measured by SAP.

Firstly, we used the MD of the tracked eye. Secondly, we correlated the VP with the MD of the two eyes combined. To calculate the severity of the two eyes combined, we used two different measures. The first one was simply the mean MD of both eyes and the second one the integrated visual field score (IVF score), as described in (22).

The IVF score is calculated by taking the maximum contrast sensitivity of each overlapping location in the visual field as the contrast sensitivity of that location. From this combined visual field, the 52 locations that make up the integrated visual field were considered in turn. A location got a score of 0 if it exhibited a measured threshold severity of ≥20 dB, scored one if it had a threshold between 10 and 19 dB, and scored 2 for a threshold below 10 dB.

Lastly, we compared the VP to the between eye difference in MD of the open and the close eye. If the difference is positive, it means that the patient watched the clips with their better eye.

#### Comparison of the Spatial Distribution of Eye Movement Features in the Visual Field Between Groups

The method used in this section is a partial replication of Crabb et al. (10), who previously used kernel principal component analysis (kPCA) in combination with a naive Bayes classifier to separate participants with and without VFD. As we did not have enough data to use machine learning, we only applied kPCA and visualized the results.

First, we defined a grid spanning the part of the visual field in which most eye movements occurred, given that the observer has an intact visual field. This area was determined by computing the median saccade amplitudes of the control group in 18 bins of 20 deg of visual angle, adding two standard deviations. To fit a rectangular grid over this area it needs to cover 21 deg half angle in the horizontal directions and 11 deg half angle in the vertical directions. The bin size was 2 deg of visual angle, as this bin size had also been applied by Crabb et al. (10). For each trial we counted the number of fixations that fell into each bin and divided the number by the maximum value of all bins so that the values range between zero and one.

We then computed the Euclidean distances between the fixation maps coming from the same video clip of different observers. The mean (*meanDist*) and the maximum (*maxDist*) of these distances were then used to construct the kernel matrices to transform the data into the feature space. We constructed one kernel of two participants i and j considering the data from all video clips using the following formula


$$\begin{array}{l} k_{ij}=e^{\frac{{-0.5\left(meanDist+maxDist\right)}^{2}}{2^{2}}} \end{array}$$

In addition, we computed one kernel per video clip of two participants i and j using the Euclidean distance (*Dist*) between the fixation maps of the respective video clips. We used the following formula:


$$\begin{array}{l} k_{ij}=e^{-Dist}. \end{array}$$

After transforming the data into feature space, we plotted the data along the first two feature axes to visualize the distances between the fixation maps of the different participants. If the spatial distribution of fixations between the two groups is different, the transformed data should form separable clusters in the new feature space.

#### Reconstructing the VFD Based on the Spatial Distribution of Eye Movement Features in the Visual Field

To test if we could reconstruct the location and shape of the VFD, we computed the average VP and the distribution of the relative fixation frequency across the visual field in a continuous manner.

To compute the distribution of VP across the visual field, we collected the fixation locations of all trials per participant and computed a fixation heat map, where each fixation was modeled as a Gaussian with a standard deviation of 1 deg. The standard deviation of the Gaussian reflects the accuracy of the eye movement data that measure the position of the eye with an error of up to 0.5 deg of visual angle. Hence, the Gaussian reflects the potential measurement error in each direction. It also reflects the size of the area in which the participants can see detail, as it is approximately the size of the foveal part of the visual field. We weighted this fixation heat map by the VP values, as well as a fixation map, where only the Gaussians were added up. We then divided the two maps, ending up with a map depicting the distribution of average VP values across the visual field.

To compute maps showing the relative frequency of fixations, with respect to the controls, we again computed heat maps of fixations as described above. We computed one map for each participant, averaged over all trials and *z*-normalized the data. For each glaucoma patient we then counted how many controls had a smaller proportion of fixations in the same location of the visual field and divided this number by the number of control participants. This way, we obtained a map showing the distribution of fixations of each patient in relation to the control group as a normalized rank value, ranging between 0 and 1.

To be better able to relate the contrast sensitivity of the visual field, as measured with the HFA to the relative frequency of fixations in the visual field of the patients, we performed the same analysis as described above with the modification to compute the relative fixation frequency in discrete bins.

We defined a grid spanning 30 deg of visual angle divided into bins of 6 deg, analogous to the test locations of the HFA SITA 30-2 visual field test, which we obtained from the patient's clinical dossier. We correlated the relative proportion of fixations in each bin with the contrast sensitivity values measured at the same location in the visual field. Then, we computed the proportion of fixations that fell into each bin averaged over all trials of each participant and compared the discrete fixation map of each patient to the fixation maps of the controls. We then obtained a relative fixation frequency map per bin.

We plotted the sensitivity value against the proportion of fixations of each location and fit a regression line through the points.

#### Statistical Testing

We assessed the median values per eye movement feature of each individual participant and compared the two groups using Mann-Whitney non-parametric testing. A *p*-value of ≤ 0.05 was considered statistically significant.

### Results

#### Basic Eye Movement Features

**Figure 1:**
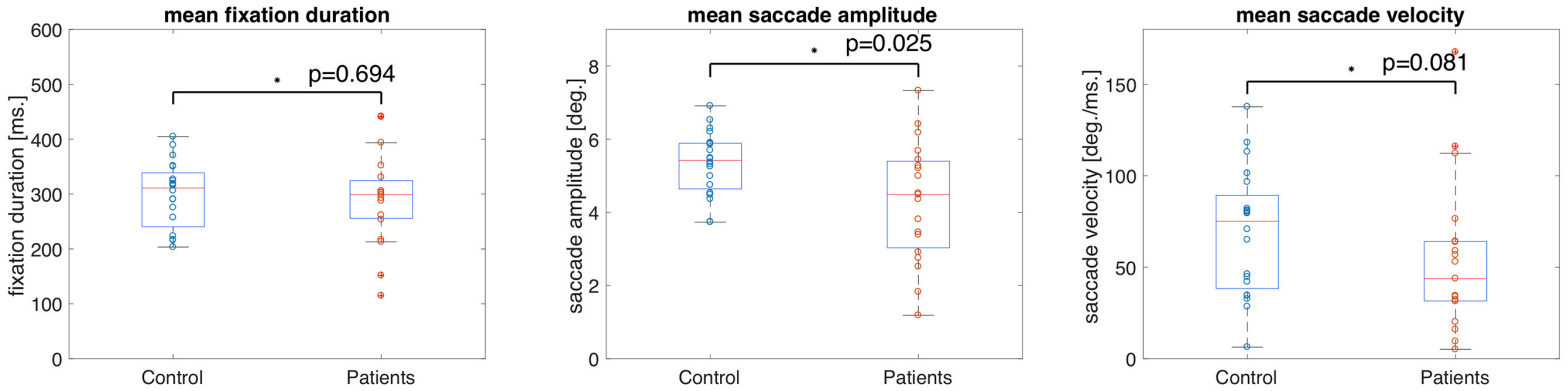
Box and whisker plots showing the means and 25–75 percentiles of three different eye movement features of glaucoma patients and controls. Circles represent the results of individual participants. The glaucoma patients showed significantly lower saccade amplitudes than the control group.

The median fixation duration was 310.96 ms (SD = 60.26 ms) for the control group and 298.94 ms (SD = 83.64 ms) for the VFD group. The Wilcoxon-Mann-Whitney test showed no significant difference between the two groups for this feature (*p* = 0.694, *z*-score = 0.39). The median saccade amplitude was 5.42 deg (SD = 0.87 deg) for the control group and 4.49 deg (SD = 1.63 deg) for the VFD group. The *p*-value of the Wilcoxon-Mann-Whitney *U*-test was *p* = 0.025 (*z*-score = 2.25), which was not significant after Bonferroni correction. The median saccade velocity was 75.14 deg/min (SD = 34.56 deg/min) for the control group and 48.81 deg/min (SD = 40.93 deg/min) for the VFD group. The Wilcoxon-Mann-Whitney test showed no significant difference between the two groups for this feature (*p* = 0.081, *z*-score = 1.74). Figure 1 shows box and whisker plots for each of the three features for patients and controls, with circles representing the values of individual participants.

#### Comparison of Directional Saccade Amplitudes Between Patients and Controls

**Figure 2:**
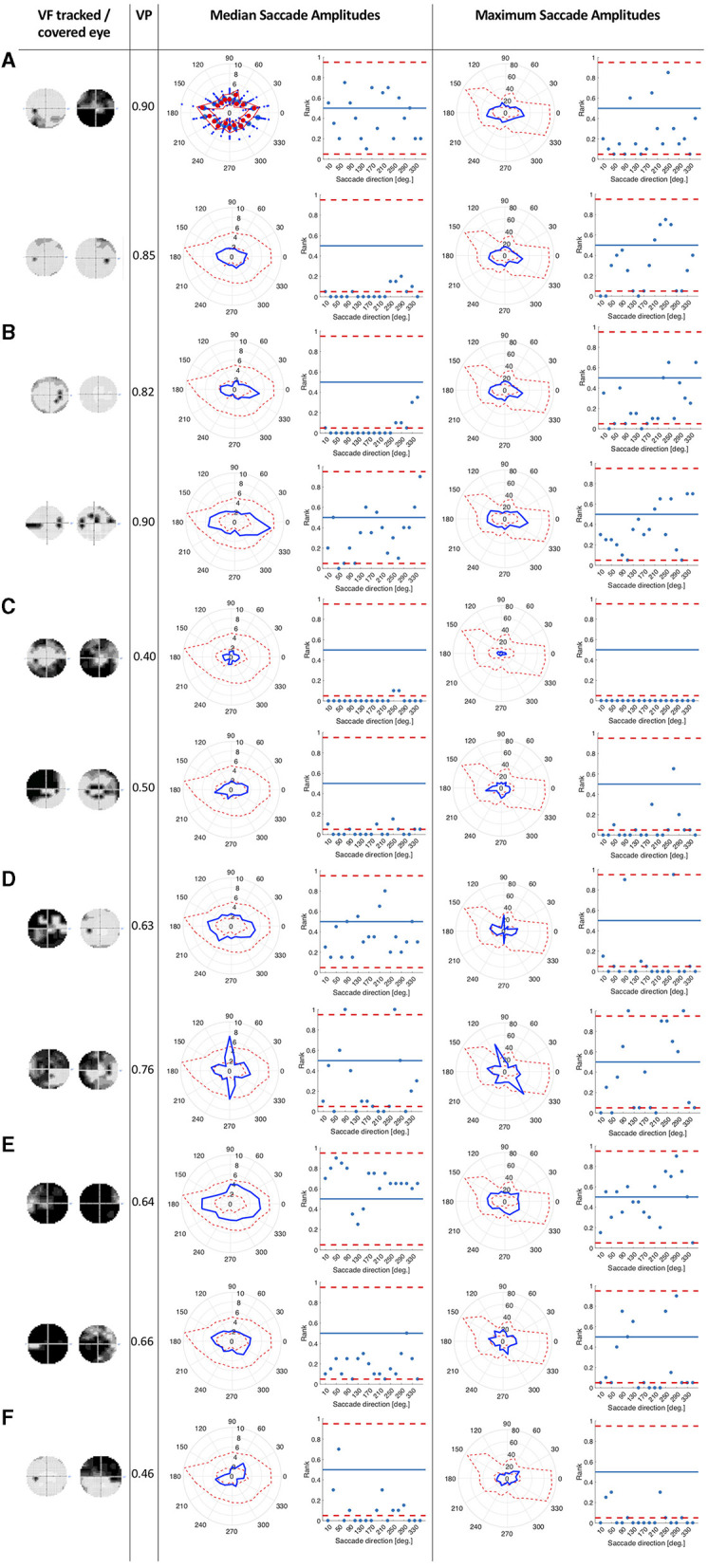
This figure consists of a series of panels, each for a group of patients with different VF characteristics. In each panel, the first column shows the visual fields of the patient as measured by SAP, with darker areas representing the less sensitive areas of the visual field. The first one is the visual field of the eye used during the experiment and the second one is the covered eye. The second column lists the average VP of the patient. The left figure in the third column shows the median saccade amplitude in 18 different directions for individual patients, connected with a blue line. For comparison, we also show the saccade amplitudes of the control group. The scattered red line represents the minimum and the maximum of the saccade amplitudes of the control participants per direction. The figure on the right shows, in terms of saccade amplitude per direction, the normalized rank of the saccades of the patient amongst those of the controls. Red lines represent the boarders at which the rank is above or below the upper and lower 2.5% of the control group. In the fourth column, the figures show the same for the maximum saccade amplitude. **(A)** Patients with peripheral visual field loss in the tracked eye. The first two patients have large VFD in their covered eyes. **(B)** Patients with a nasal arc or other small VFD, which were closer to the center than the VFD of the patients in the first group. **(C)** Patients with VFD that approximate tunnel vision in the tracked eye. **(D)** Patients with large VFD in various parts of the visual field. **(E)** Patients who are almost blind. **(F)** Patient, with an intact visual field of the tracked eye.

We tested whether the location of the VFD has an influence on saccade amplitudes by comparing the saccade amplitudes in 18 different directional bins of each patient and the control group. We hypothesized that the saccade amplitudes that patients made toward directions in which a VFD was located would differ from the saccade amplitudes of the control group toward the same location. We tested this hypothesis by computing the ranks of the directional saccade amplitudes of each patient with VFD compared to the control group. Figure 2 shows in which directions the median and maximum directional saccade amplitudes of representative glaucoma patients differ from those of the control group. It shows their ranks relative to the saccade amplitudes of the control group. As a reference, it also shows the average VP of this patient over all trials and the visual field sensitivity of both eyes.

Patients with peripheral VFD in the tracked eye, as shown in panel 2a, mostly showed similar saccade amplitudes to the control group. However, the second patient in panel 2a displayed lower median saccade amplitudes than the control group in several directions, with ranks of those saccade amplitudes being below the 5% lowest saccade amplitudes of the controls.

The first patient shown in panel 2b showed smaller median saccade amplitudes than the control group, except in the directions where the VFD in the tracked eye was located. The second patient in panel 2b showed similar median saccade amplitudes to the control group, making the largest saccades toward the area of the nasal arc.

The patients with tunnel vision in the tracked eye, shown in panel 2c showed lower median and maximum saccade amplitudes compared to the control group in all directions.

The first patient in panel 2d, with a large VFD in the tracked eye, that also reached the center of the visual field, showed similar median saccade amplitudes to the control group, but reduced maximum saccade amplitudes. The second patient in panel 2d showed similar median and maximum saccade amplitudes as the control group, except in the vertical directions. The upwards and downwards saccade amplitudes are larger than those of the control group.

Panel 2e shows two patients, who were almost blind in their tracked eye. They showed similar median and maximum saccade amplitudes to the controls. The first one shows median saccade amplitudes above the average of the control group, while the second one shows median saccade amplitudes below the average of the control group. But both of them remain within the 95% confidence interval of the control group.

Panel 2f shows the patient who performed the experiment with their intact eye. This patient displayed slightly shorter median and maximum saccade amplitudes than the controls, especially toward the top-left and lower right quadrant of the visual field.

#### Viewing Priority

We compared the median VP of patients and controls to test if the scan paths of the two groups differed when viewing the different video clips using a Wilcoxon Mann Whitney test.

**Figure 3:**
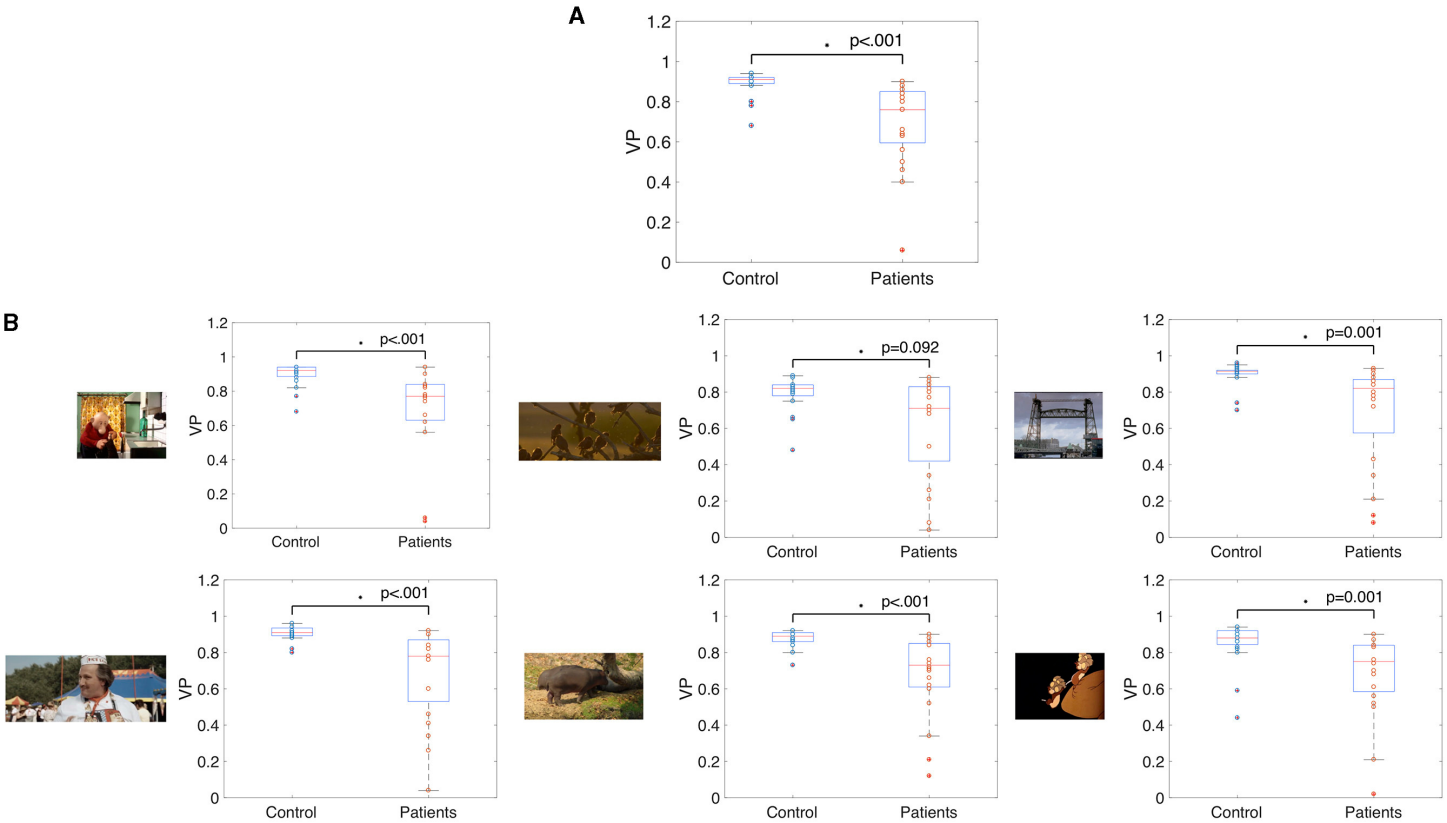
Box and whisker plots showing the median VP values of glaucoma patients and controls **(A)** averaged over fixations of all trials and **(B)** averaged over fixations of single video clips. The median VP of the two groups differed significantly (*p* < 0.05) in all but one movie clip.

The median VP value of the control group was 0.91 (SD = 0.06) and the median VP of the glaucoma group was 0.76 (SD = 0.21). The group medians in VP of the two groups are significantly different when taking the average VP over all trials (*p* < 0.001, *z*-score = 4.34). Figure 3A shows these results as a boxplot. When computing the average VP per trial we found that the group median of the glaucoma patients was significantly lower (*p* < 002) for 22 out of 28 movie clips. Boxplots showing the range of VP values and the group medians for the control group and the glaucoma group of exemplary video clips can be seen in Figure 3B.

**Figure 4:**
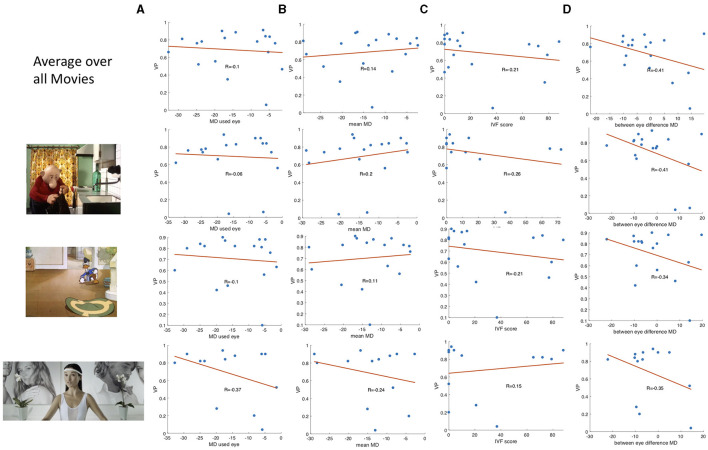
Boxplots showing **(A)** the correlation between the VP value and the MD value of the measured eye of the participants with VFD, **(B)** the correlation of the IVF score and the VP, and **(C,D)** the correlation of the difference between the MD value of the measure.

While the group median VP of the patients was reduced compared to the control group, Figure 3 also shows that there is a high variance in VP among the patients with VFD. We correlated the average VP of the patients with VFD with measures indicating the severity of the visual field damage obtained by SAP to determine which factors could potentially influence this variability. Figure 4 shows the correlation of the VP values with the MD of the tracked eye, the IVF score and the difference of the MD of the two eyes. We found no correlation between the VP value averaged over all trials and the MD of the tracked eye (Pearson's *r* = 0.03). We found a weak correlation between the VP values averaged over all trials and the IVF score (Pearson's *r* = 0.11) and we found a moderate correlation between the VP values averaged over all trials and the difference in MD between the measured and the covered eye (Pearson's *r* = 0.39).

When averaging the VP value over data of individual video clips, the correlation coefficients varied strongly between video clips. Some representative examples of data from different video clips are shown in Figure 4B.

#### Spatial Distribution of Eye Movement Features in the Visual Field Used to Distinguish Between Patients and Controls

**Figure 5:**
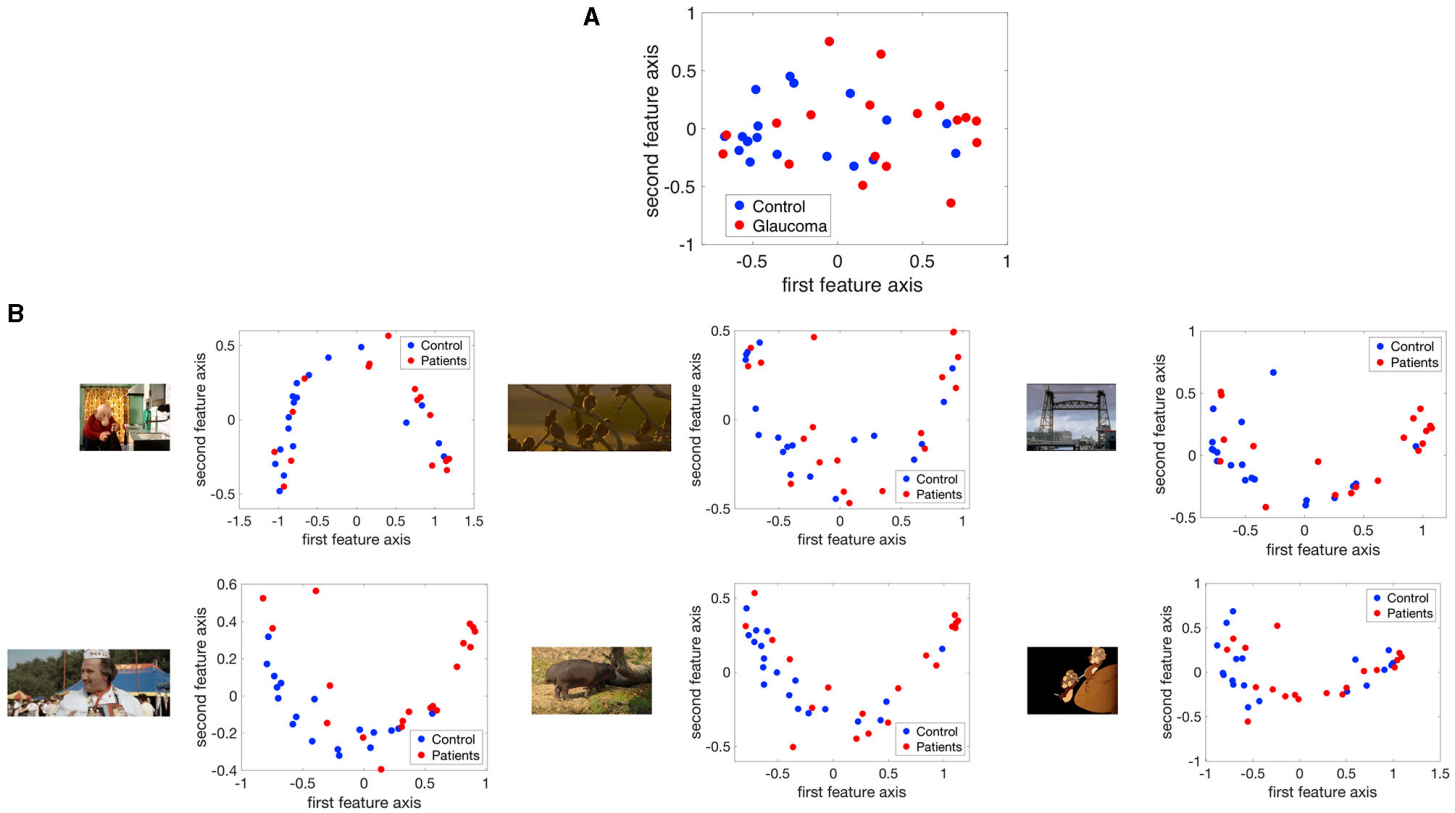
The fixation maps of each participant projected onto the two axes of the high dimensional kernel space with the highest covariance. We used **(A)** the mean and the maximum Euclidean distance between all fixation maps of each participant for the kernel and **(B)** the distance between fixation maps of individual video clips. These are representative examples.

Figure 5 shows the projection of the fixation maps onto the first two most significant feature axes from the kPCA using the data of all video clips and representative examples of using data from individual video clips. kPCA transforms the original data (maps of the visual field) onto a new space, in this case using the distances between individual visual field maps for the kernel matrix. If patients with VFD can be separated from controls, the two groups should form two separable clusters in this new feature space.

When performing the kPCA considering all trials of each subject, the first two significant feature axes accounted for 25% of the variance in the data. Figure 5A shows that the two groups projected onto these feature axis overlaps, while more of the data points representing participants of the glaucoma group cluster on the right side of the plot, where the first feature is positive. The majority of the data points representing participants of the control group is located on the left side of the plot, where the first feature is <0. When performing the kPCA per video clip, we found that the first two feature axes together explained between 28 and 36% of the variance, depending on the video clip. The variance explained of the third feature was much lower with around 7%. Figure 5B shows that the two groups tend to separate along the first feature axis when we project data of individual video clips. However, the data of some video clips.

#### Spatial Distribution of Eye Movement Features in the Visual Field Used to Reconstruct the VFD

**Figure 6:**
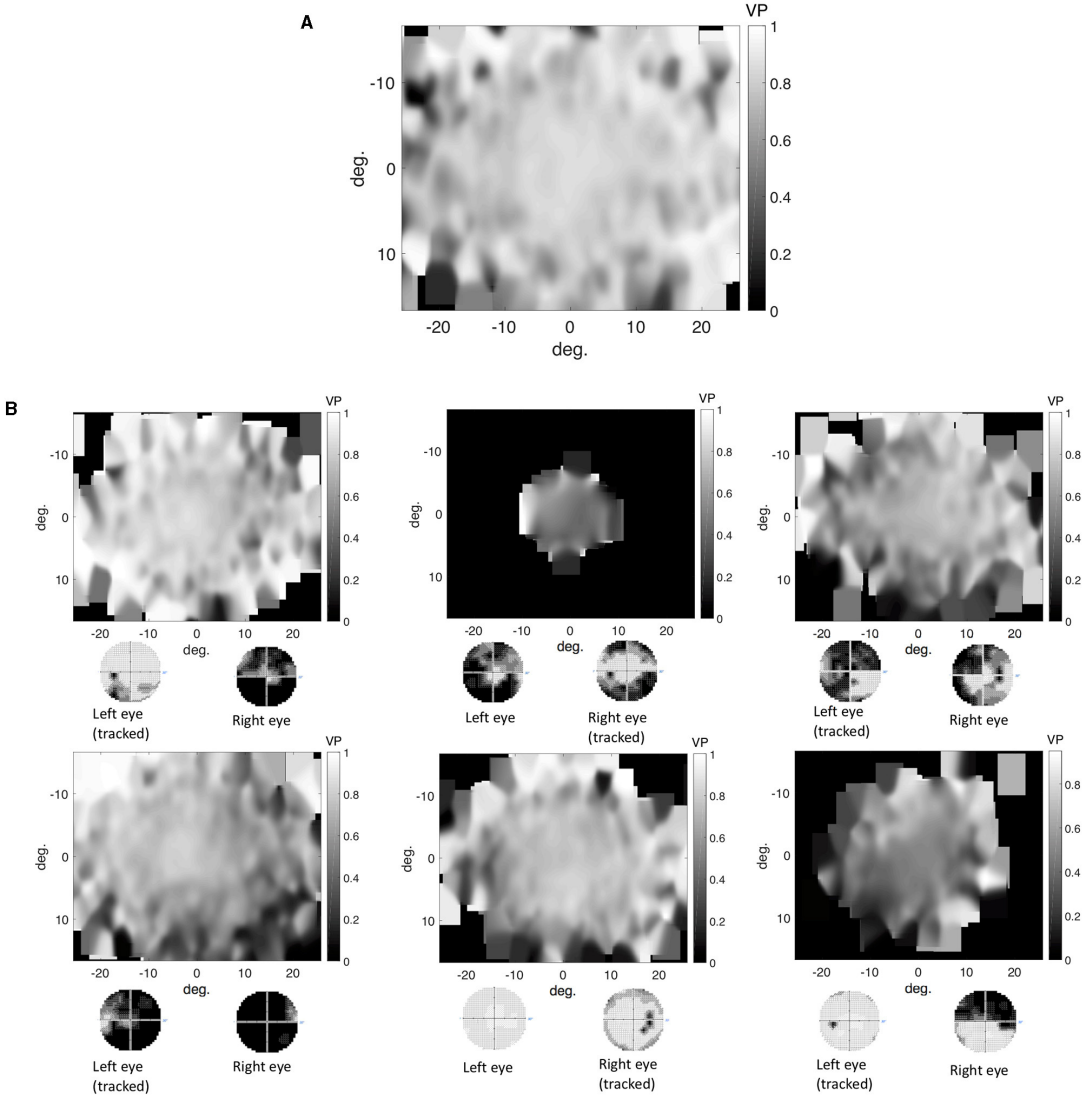
**(A)** Representative example of the distribution of VP across the visual field of a control participant. **(B)** Representative examples of VP maps of participants with different types of VFD. The distribution of the VP usually does not correspond to the sensitivity values of the visual field, except for the participant with tunnel vision, which is due to the fact that they never direct their gaze toward the periphery and therefore have a VP of 0 in these areas.

We found that in the control group the intact visual field is correlated with an evenly distributed, high VP across the visual field. Individual glaucoma patients showed a large variability in the distribution of VP across the visual field. Both findings are shown in Figure 6, which depicts the distribution of VP across the visual field of one representative control participant and several glaucoma patients. The distribution of the VP depended to some degree on the location and severity of the VFD. Patients with large peripheral VFD displayed a lower VP in the periphery of their visual field. In other glaucoma patients, the distribution of the VP does not correspond to their VFD. Figure 6B shows some representative examples.

**Figure 7:**
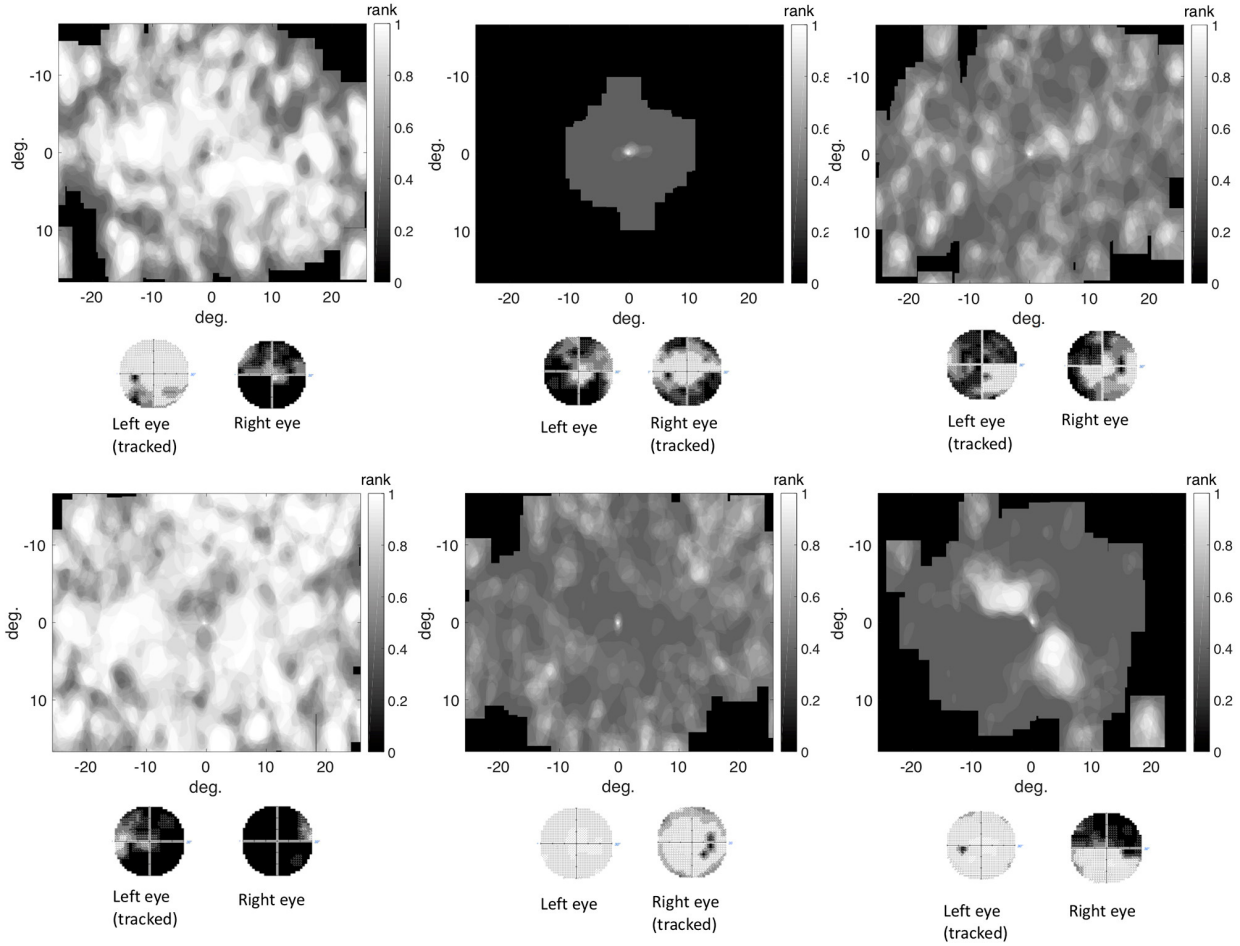
The differential fixation maps of the same patients. A value higher (lower) than 0.5 means that the patient made more (less) eye movements toward a part of the visual field than the control group.

In addition, we tested if the distribution of fixations across the visual field of glaucoma patients, differed systematically from the distribution of fixations of the control group, in such a way that it correlates with the location of the VFD. If glaucoma patients directed their gaze significantly more or less frequently toward damaged areas of the VFD, the distribution of fixation frequency could be used to localize VFD. Figure 7 shows that, similar to the distribution of the VP, the different glaucoma patients showed a lot of variability in the distribution of fixations across the visual field. Patients with a large peripheral VFD made significantly fewer eye movements toward the periphery than the control group, which led to a relative fixation frequency map that matched the sensitivity of their visual field. In other glaucoma patients, the relative distribution of fixations across the visual field did not correspond to the distribution of sensitivity.

#### Correlation Between the Measured Sensitivity by the HFA and the Fixation Frequency

**Figure 8:**
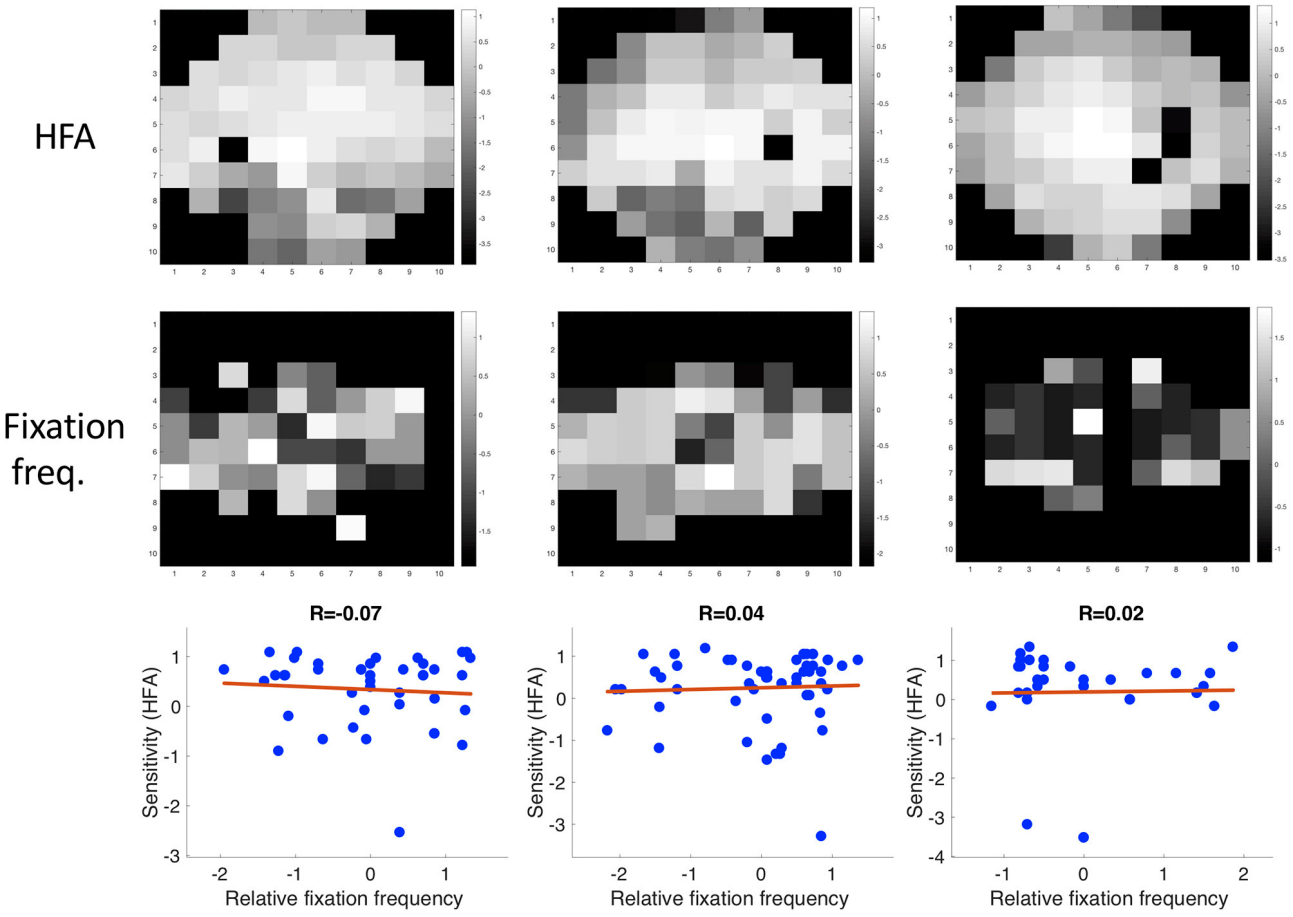
Three examples showing the correlation between sensitivity and the relative frequency of fixating in a discrete region in the visual field.

There was no correlation between the location of the VFD and the frequency in which different regions of the visual field were fixated. The mean correlation coefficient between the relative fixation frequency and the sensitivity values was: Pearson's *r* = 0.160 (minimum: Pearson's *r* = −0.138, maximum: Pearson's *r* = 1.289). This is also shown in Figure 8, where we plotted the relative fixation frequency and the sensitivity values in discrete regions of 6 deg over a visual field spanning 30 deg half angle of representative example patients.

## Experiment 2: Binocular Eye Movements Under Free-Viewing Conditions of Glaucoma Patients Compared to Those of Normal Sighted Observers

### Methods

#### Summary

In this experiment, we analyzed a data set collected at City College, London, published by (15) using a similar pipeline as in experiment 1. In this experiment, eye movement data was collected for glaucoma patients and age matched controls who each viewed three different video clips with both eyes.

#### Participants

We used the data set published by Asfaw et al. (available at https://doi.org/10.5281/zenodo.1156863). They had collected data of 44 people with glaucoma recruited *via* the ophthalmology clinics at Moorfield's Eye Hospital NHS Foundation Trust, London using an Eyelink 1000 eye tracker (SR Research Ltd., Ontario, Canada). All patients had an established clinical diagnosis of chronic open angle glaucoma (COAG) for at least 2 years and were between 50 and 80 years of age. They had different VFD in each eye. More details concerning the state of their visual field can be found in the publication of the data set. Thirty-two healthy people (controls), of a similar age to the patients, were recruited from the City University London Optometry Clinic. The dataset provides ophthalmic information on each participant (visual acuity, contrast sensitivity per location of each eye, and visual field loss as MD value), raw eye movement data, as well as the eye movement features (fixation locations, fixation duration, start and end points of saccades, saccade amplitude and peak velocity, and pupil area) recorded while they viewed movies. We used the processed eye movement data in the following analysis.

#### Stimulus Materials

Participants viewed three different video clips presented on a 54 cm monitor (Iiyama Vision Master PRO 514, Iiyama Corporation, Tokyo, Japan) at a resolution of 1,600 by 1,200 pixels (refresh rate 100 Hz). The first video clip was part of an entertainment program called Dad's Army (BBC Television) 309 s long and covered the full screen (subtending a half-angle of 20.3 deg by 14.9 deg). The second film clip was taken from “The History Boys” (Twentieth Century Fox) and 200 s long. The third clip was taken from a sports program “2010 Vancouver Winter Olympics Men's Ski Cross” (BBC Television) and 436 s long. The last two clips were recorded at a 16:9 ratio, therefore they contained black rectangles at the top and bottom of the screen. They subtended a half-angle of 17.3 deg by 10.6 deg.

#### The Integrated Visual Field (IVF)

Participants viewed the movies with both eyes open. This means that they viewed the video clips with their integrated visual field (IVF). We computed the sensitivity value of each location in the IVF by merging the provided sensitivity values of corresponding locations in each eye. The sensitivity values had been measured with a Humphrey Field Analyzer (HFA; Carl Zeiss Meditec, CA, USA), with a standard 24-2 grid and the Swedish Interactive Testing Algorithm (SITA). We used a best location approach to determine the sensitivity of each location in the IVF, meaning that we selected the highest sensitivity value among the two eyes as the sensitivity of the IVF (23).

We used the IVF as the reference for visual field sensitivity. For each glaucoma patient, we calculated the integrated visual field score (IVF score) as defined by (22). The IVF score is a summary measure of the state of the IVF, with a higher IVF indicating more severe damage to the visual field.

### Eye Movement Data Analysis

#### Fixations and Saccades in Visual Field Coordinates

Analogous to experiment 1, saccades were defined using a velocity threshold of 30 deg/s and an acceleration threshold 8,000 deg/s^2^ using the Eyelink's built-in algorithm. All other eye movement data was classified as a fixation. The processed eye movement data (fixation location, saccade start and end points) were provided as x-y coordinates on the screen, where the origin of the screen was the top left corner.

We defined the locations of the saccade start and end points and the fixations in the same way, as described in experiment 1.

#### Basic Eye Movement Features

To get a first impression of differences in eye movement behavior between the two groups, we computed the mean fixation duration, the mean saccade amplitude and the mean saccade velocity over all three video clips. We compared the two groups using a Wilcoxon-Mann- Whitney *U*-test. As in experiment 1, Bonferroni corrected *p*-value of 0.013 was regarded as significant.

#### Comparison of Directional Saccade Amplitudes of Glaucoma Patients and Controls

To compare the directional saccade amplitudes between individual patients and controls, we performed the same analysis as in experiment 1, with the small difference that we compared the saccade amplitudes to the IVF instead of the visual field of one eye. First, we computed the median and maximum saccade amplitudes of each participant in 18 different directional bins. We compared the saccade amplitudes per bin of each patient to those of the controls. To evaluate if patients with similar kinds of VFD in their IVF showed similar saccade amplitudes, we defined four different groups of patients, according to size and location of the VFD. The first one included patients with small peripheral VFD, as occur in early stages of glaucoma. The second one included patients with a VFD in the upper half of the visual field. The third group consisted of patients with large peripheral VFD (tunnel vision), as occur in late stages of the disease. The fourth group consisted of patients with a VFD in the central part of the visual field. For each patient's median and maximum saccade amplitudes, we computed their ranks among the saccade amplitudes of the controls in the same directional bin and divided it by the number of control participants, so that it ranged between zero and one. Again, we did not perform any statistical inference tests, as we only had a small groups of participants with a similar type of VFD in their IVF.

#### Viewing Priority

We extracted the fixations of each participant from the pre-processed data and computed the viewing priority (VP) for each of them (12, 16). As in experiment 1, we used fixations made by the control group both as the reference and as the random set of fixations.

We averaged the VP value from all fixations of each trial for each participant and compared the two groups using a Wilcoxon-Mann-Whitney *U*-test. As participants had watched three video clips, in this experiment, we corrected for three repeated measures. A Bonferroni corrected *p*-value of 0.017 was regarded as significant. In addition, we computed the correlation coefficient of the VP and the integrated visual field score (IVF score) as defined by (22) of each glaucoma patient. We hypothesized that a more damaged IVF would result in a lower VP.

#### Comparison of the Spatial Distribution of Eye Movement Features in the Visual Field Between Groups

1. Replication and extension of the analysis of Crabb et al. (10):

First, we transformed all eye movement features into visual field space. Following Crabb et al. (10), we computed “saccade maps” on a grid of 12 by 10 deg half angle that was subdivided into 2x2 degree bins. The grid excluded the central four bins, thereby ignoring fixations that occurred after a very short saccade. We computed the proportion of saccades that ended in each bin. In addition to replicating their analysis, we extended it by also computing “fixation distribution maps” and “VP maps.” To compute the fixation distribution maps, we also included the proportion of fixations that followed a saccade into the central four bins. For the VP maps, we computed the average VP in each bin.

We transformed the saccade and fixation maps into a high dimensional feature space using kernel PCA with a Gaussian kernel, where the distance between any two participants i and j was determined with the following kernel: $k_{ij}=e^{\frac{{-0.5\left(meanDist.+maxDist.\right)}^{2}}{0.2^{2}}}$.

For the VP maps we used the following kernel: $k_{ij}=e^{\frac{{-0.5\left(meanDist.+maxDist.\right)}^{2}}{2^{2}}}$. The Gaussian distribution has a larger variance, because the values in these maps also had a larger variance.

*MeanDist* and *maxDist* were the mean and maximum of the Euclidean distances of the respective feature maps. In addition, we again performed the kPCA on each of the trials separately, computing the Euclidean distance (*Dist*) between the fixation maps of the respective video clips, using the following kernel: $k_{ij}=\;e^{-Dist.}$.

For each movie, we visualized the first two dimensions of the transformed data in the kernel feature space. Finally, we used the first five dimensions of the projected data as input to train a naïve Bayes classifier. The classifier was trained and validated using 10-fold cross validation, training on 90% of the data and testing on the remaining 10%.

2. Application of the method used in experiment 1

In experiment 1, we had used a grid that included 95% of the saccade end points. To achieve the same on this data set, we calculated the mean saccade amplitude plus two standard deviations in the horizontal (left and right) and vertical directions (up and down) in the control group. To create a symmetric grid, the maximum value of these four values was used. This resulted in an 8 × 10 bin grid of 16 by 20 deg of visual angle, with bin size set to 2 deg horizontally and vertically. We then computed the number of fixations that fell into each bin per video clip and divided it by the maximum value in the grid. To compare this approach to the analysis in experiment 1, we applied the kPCA and visualized the first two feature axes in kernel space. We performed kPCA using the same kernels as in experiment 1. We first computed the Euclidean distances between their fixation maps and used the mean distance (*meanDist*) and the maximum distance (*maxDist*) between their fixation maps to construct the following kernel between the participants i and j: $k_{ij}=e^{\frac{{-0.5\left(meanDist+maxDist\right)}^{2}}{2^{2}}}$.

In addition, we applied the kPCA to the fixation maps of each video clip individually by first computing the Euclidean distance between the fixation maps of the respective video clip. Two participants i and j were then separated using the following kernel: $k_{ij}=\;e^{-Dist.}$.

#### Reconstructing the VFD Based on the Spatial Distribution of Eye Movement Features in the Visual Field

We tested whether we could use the spatial distribution of the VP and the relative fixation frequency to reconstruct the VFD in the IVF of the patients. To be able to compare the two eye movement features to the sensitivity of the IVF, as measured by the combination of the 24-2 visual fields of the two eyes, we computed VP maps of the central 24 deg of the visual field, divided into 6 × 6 deg bins. To construct VP maps, we computed the average VP of all fixations that fell into a bin.

To construct the maps depicting the relative frequency of fixations, for each participant we summed up all fixations that occurred in one bin and divided them by the total number of fixations. We then compared the proportion of fixations that fell into each bin of one glaucoma patient to the proportions of fixations that fell into the same bin of each of the control participants. We counted how many controls had a smaller proportion of fixations in the same location of the visual field than the respective patient and divided this number by the number of control participants. This resulted in the proportion of fixations per location of that patient being depicted as a rank, ranging between 0 and 1. A value close to 1 meaning that they fixated this area more frequently than the controls.

### Results

#### Basic Eye Movement Features

**Figure 9:**
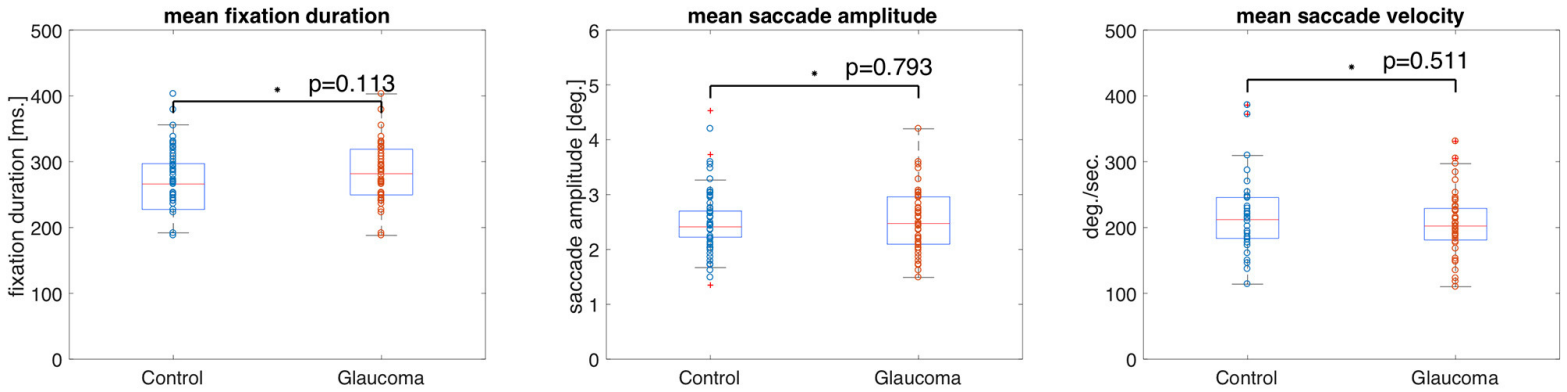
Box and whisker plots showing the mean and variance of four different eye movement features for the control and glaucoma groups. The circles represent the values of the individual participants. The group means did not differ significantly.

Figure 9 shows that the group medians did not differ significantly on any of the basic eye movement features we investigated. The median fixation duration of the control group was 266 ms (SD = 44.92 ms) and 281.75 ms (SD = 45.48 ms) for the patients with VFD. The Wilcoxon-Mann-Whitney test did not show a significant difference in fixation duration between the two groups (*p* = 0.113, *z*-score = −1.584). The median saccade amplitudes of the control group was 2.41 deg (SD = 0.57 deg) and 2.48 deg (SD = 0.58 deg) for the patients with VFD. The Wilcoxon-Mann-Whitney test did not show a significant difference in saccade amplitude between the two groups (*p* = 0.793, *z*-score = −0.263). The median saccade velocity of the control group was 212 deg/s (SD = 60.07 deg/s) and 202.25 deg/min (SD = 47.77 deg/min) for the patients with VFD. The Wilcoxon-Mann-Whitney test did not show a significant difference in saccade velocity between the two groups (*p* = 0.511, *z*-score = 0.658).

#### Comparison of Directional Saccade Amplitudes Between Patients and Controls

If the directional saccade amplitudes of a patient are influenced by the location of the VFD, we should find that the median and maximum saccade amplitudes, in directions where a VFD is located, deviate from those of the control group.

**Figure 10:**
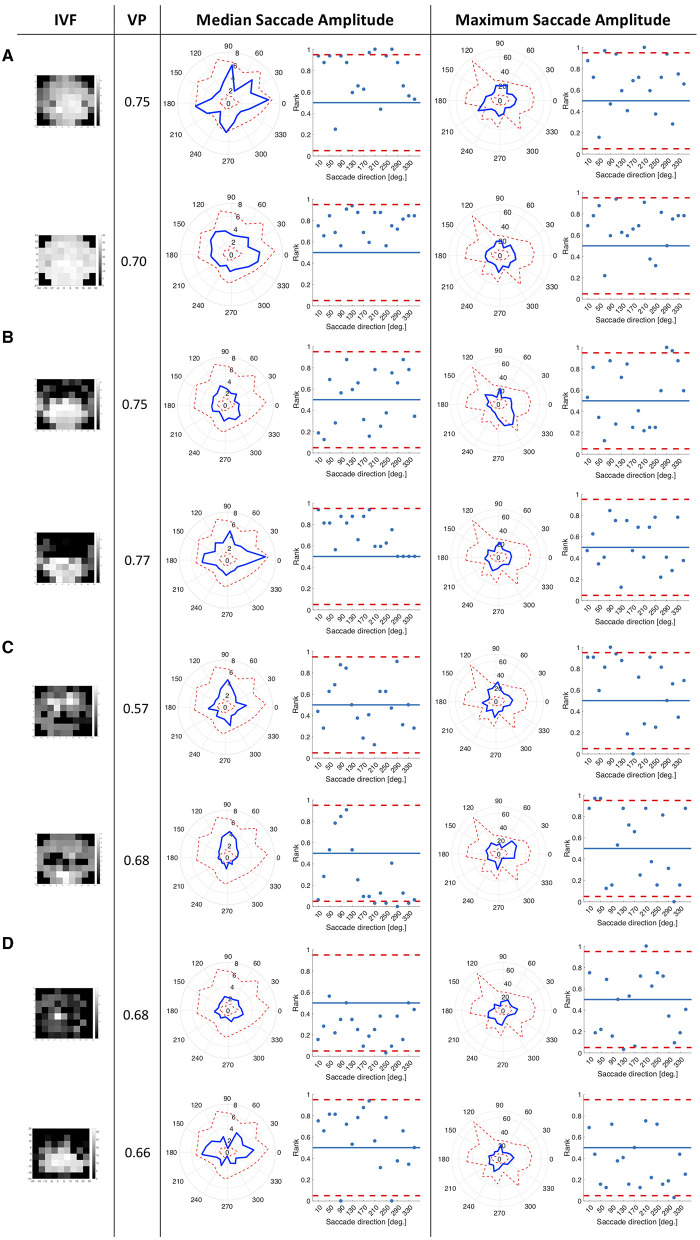
The first figure shows the state of the IVF of each patient, with darker areas representing the less sensitive areas of the visual field. The second column shows the VP of this patient averaged across all trials that they performed. The third column shows the median saccade amplitudes in 18 directional bins of each patient, represented by the blue line. The range of the saccade amplitudes by the control group is represented by the scattered red line, showing the minimum and the maximum of the saccade amplitudes of the control group, per direction. The second figure in this column shows the normalized rank that the saccade amplitude of the patient occupies among the saccade amplitudes of the controls per direction, with the red lines representing boarders at which the rank is above or below the upper and lower 2.5% of the control group. The fourth column shows the same as the third column for the maximum saccade amplitudes **(A)** Examples of median and maximum saccade amplitudes of four different patients, who had peripheral VFD in their IVF. **(B)** Examples of patients with VFD in the upper part of the visual field. **(C)** Patients with a VFD which also reached the central part of the visual field. **(D)** Examples of patients with large VFD in the periphery of the visual field. While the first and third patient in this panel showed median and maximum saccade amplitudes which were close to those of the controls, the second one showed high median saccade amplitudes toward the most intact, but also toward the most damaged area of the visual field.

Overall, the large majority of the patients' median and maximum saccade amplitudes remained within the range of those of the control group, as can be seen in Figure 10.

Examining individual patients in more detail, participants shown in Figure 10A, showed high median saccade amplitudes compared to the control group, in some directions higher than 95% of the participants in the control group. They also showed maximum saccade amplitudes which were higher than those of most of the participants in the control group in some directions.

Panel 10b shows examples of patients with a VFD in the upper part of the visual field. They made similar saccade amplitudes compared to the control group.

Panel 10c shows patients with a VFD covering the central part of the visual field. Their median upwards saccade amplitudes were larger than their downwards or sidewards median saccade amplitudes.

Panel 10d shows two patients with big VFD in the periphery. They showed median and maximum saccade amplitudes outside the 95% confidence interval in some directions.

#### Viewing Priority

**Figure 11:**
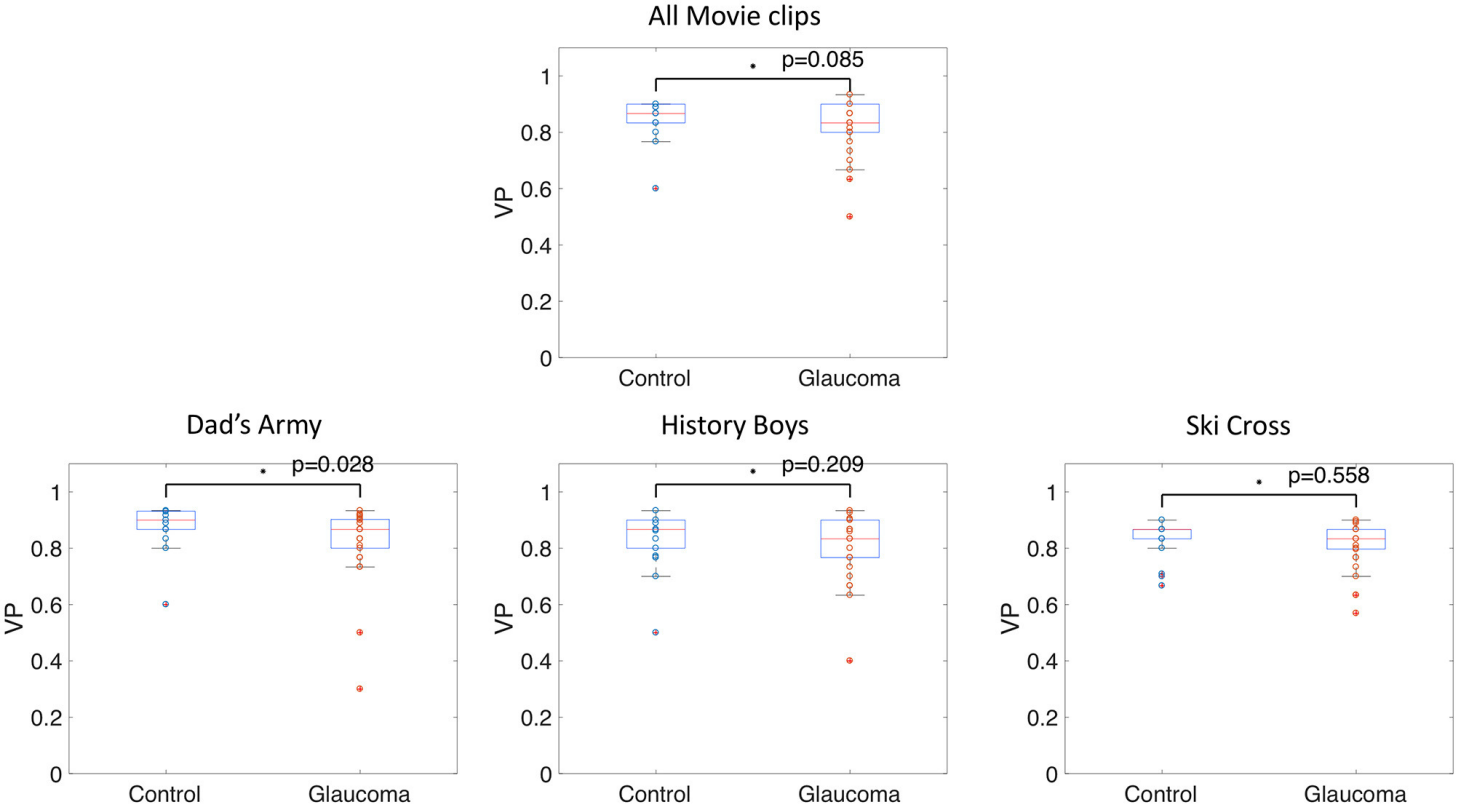
Box and whisker plots showing the group means of VP per movie clip. The circles represent the VP value of each participant.

A significant difference in VP between the two groups would indicate that their fixation locations while viewing the same scene often differ. Figure 11 shows the median VP value of the control groups was 0.87 (SD = 0.06) and of the glaucoma patients was 0.83 (SD =0.08). The Wilcoxon-Mann-Whitney test did not show a significant difference (*p* = 0.085, *z*-score = −1.723). When calculating the average VP values per movie clip, we found that the median VP value of the control group was 0.9 (SD = 0.07) and of the glaucoma patients was 0.87 (SD =0.13) for the movie clip “Dad's Army.” The median VP value of the control group was 0.87 (SD = 0.09) and of the glaucoma patients was 0.83 (SD = 0.11) for the movie clip “History Boys.” The median VP value of the control group was 0.87 (SD = 0.09) and of the glaucoma patients was 0.83 (SD = 0.08) for the movie clip “Ski Cross.” We did not find a significant difference in the group medians of patients and controls in VP, when averaging over the data collected during the video clips “Dad's Army” (*p* = 0.028, *z*-score = −2.914), “History Boys” (*p* = 0.209, *z*-score = −1.257), and “Ski Cross” (*p* = 0.558, *z*-score = −0.586).

**Figure 12:**
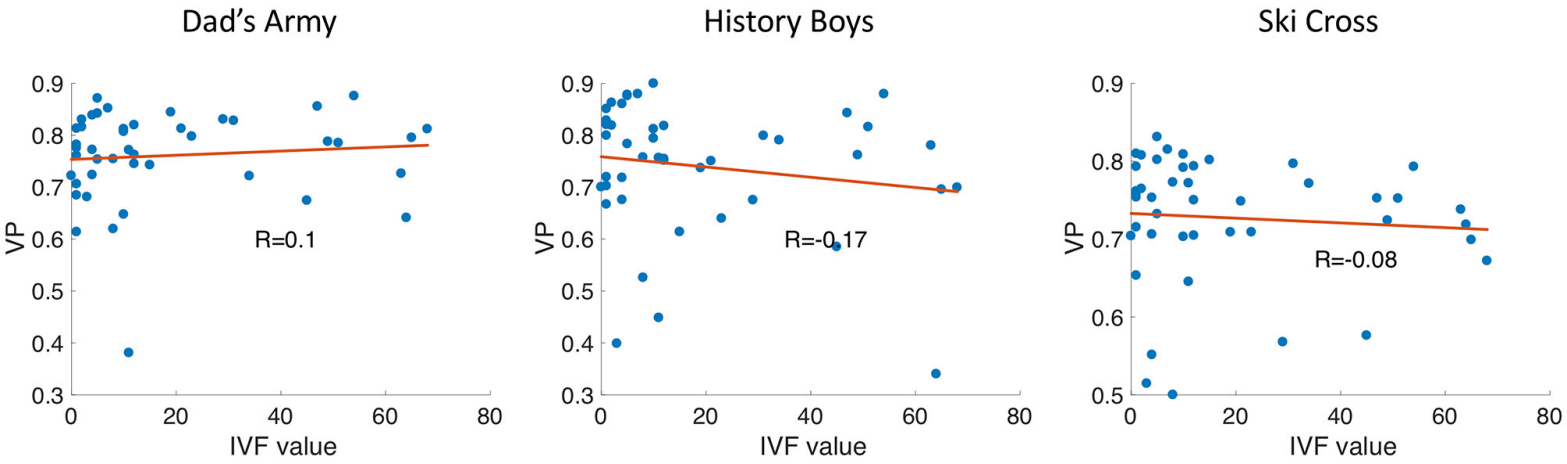
Scatter plots showing the VP vs. the IVF score of each participant. *R* is the correlation coefficient.

Figure 12 shows the IVF score plotted against the median VP of each glaucoma patient. As the IVF score increases with a more severely damaged visual field, we would observe a negative correlation of VP and IVF score if the VP value decreased with disease severity. When we compare the IVF score against the median VP of each patient from the video clip “Dad's Army” we find no correlation (Pearson's *r* = 0.04). We find a very small negative correlation when we compare the patients' IVF against the VP from the video clips “History Boys” (Pearson's *r* = −0.18) and “Ski Cross” (Pearson's *r* = −0.17).

#### Spatial Distribution of Eye Movement Features in the Visual Field Used to Distinguish Between Patients and Controls

1. Replication

**Figure 13:**
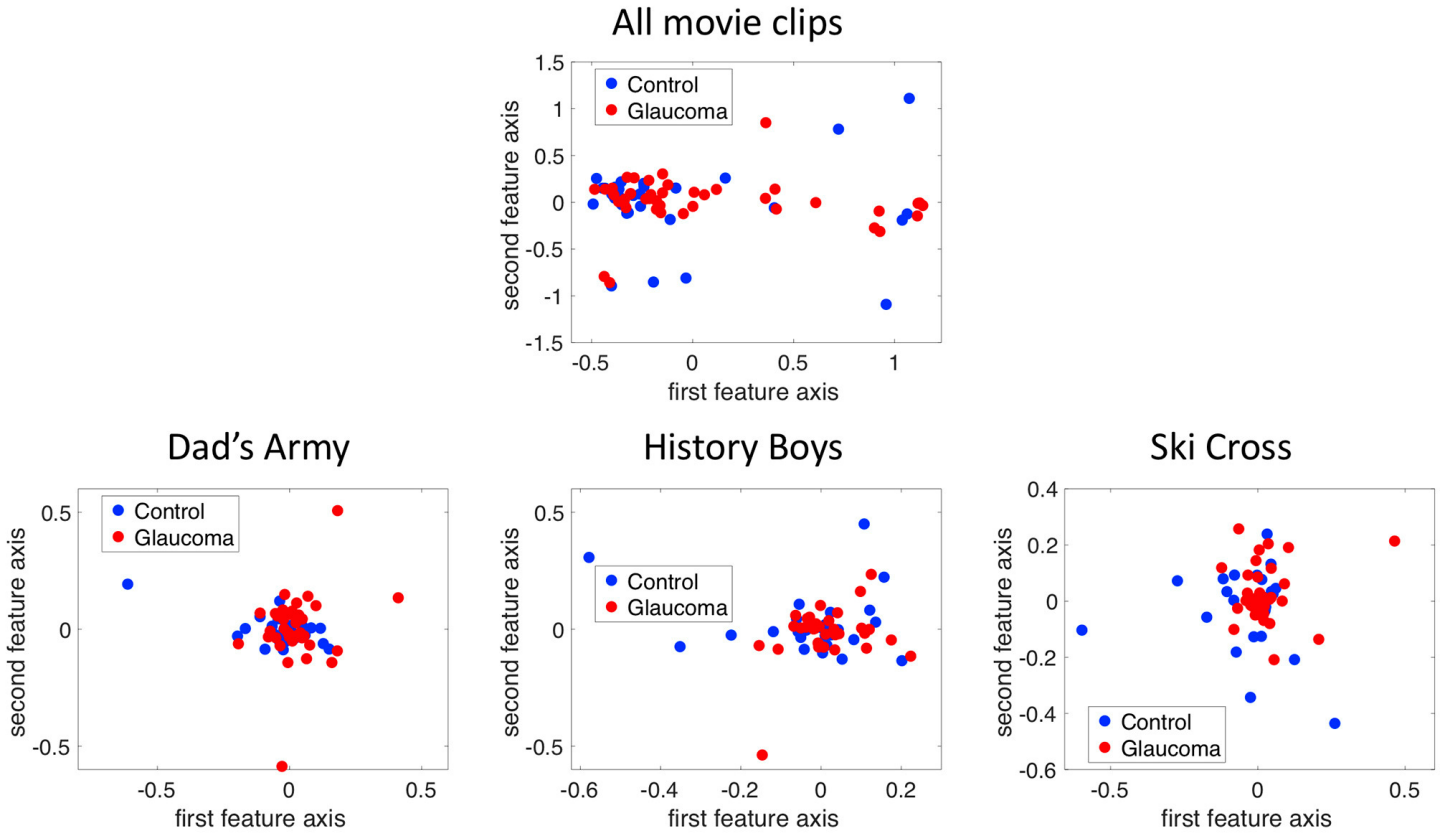
Projection of the 12 by 10 bin saccade maps onto the first two principal axes in the kernel space using the combined data of all three trials and the data of each trial separately.

If the two groups formed separable clusters in the kernel space, it would be an indicator that they could be separated in this high dimensional feature space. We found that the two groups do not separate very well along the first two feature axes as can be seen in Figure 13, which shows the data of the saccade maps projected onto the first two significant feature axes of the kPCA. We found that the first five dimensions in kernel space explain 25% of the variance in the data for the saccade maps if we use all three video clips. When performing the same analysis with the fixation and VP maps, the transformation of the data into the feature space leads to similar results (see Figures 13B,C).

Table 3 shows the classification accuracies of the naive Bayes classifier using the first five significant feature axes after 10-fold cross validation.

**Table 3:** Accuracy of the naïve Bayes classifier for each of the maps.

**Eye movement feature**	**All trials**	**Dad's army**	**History boys**	**Ski cross**
Saccade map	54.7% (36.8 – 63.2%)	50.0% (31.6 – 63.2%)	46.3% (26.3 – 63.2%)	50.0% (36.8 – 63.2%)
Fixation map	57.9% (47.4 – 73.7%)	54.2% (42.1 – 68.4%)	62.6% (47.4 – 79.0%)	50.5% (31.6 – 68.4%)
VP map	52.6% (31.6 – 68.4%)	48.4% (36.8 – 57.9%)	47.4% (31.6 – 57.9%)	49.5% (26.3 – 68.4%)

*Using data of all three trials or each trial separately. In brackets, we show the minimum and maximum accuracy achieved in the 10-fold cross validation*.

2. Application of the method used in experiment 1

**Figure 14:**
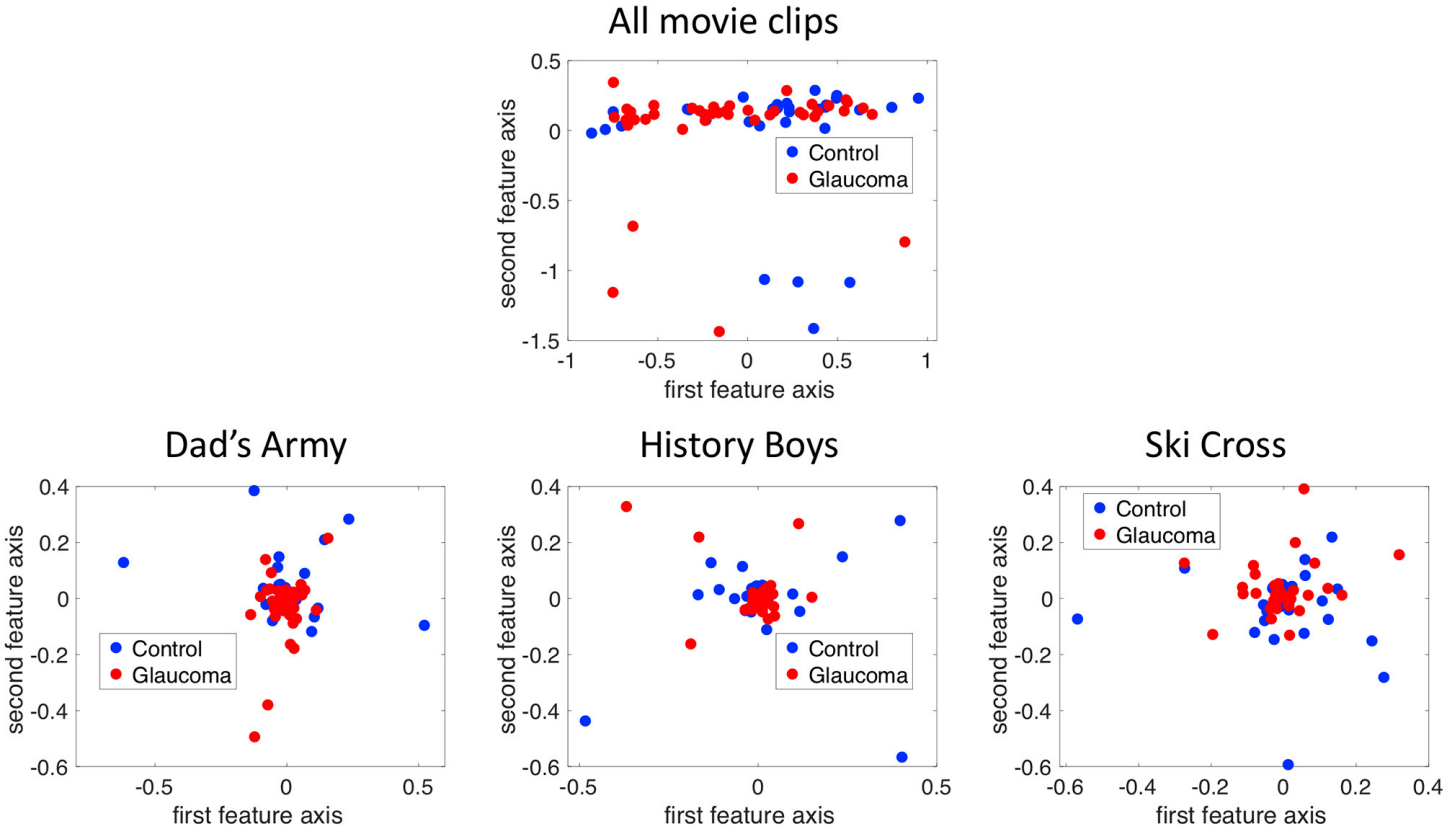
Projection of the 8 by 10 bin fixation maps onto the first two principal axes in the kernel space using the combined data of all three trials and the data of each trial separately.

In addition, we projected the eight by 10 bins fixation maps onto the first two principal components using kPCA on the data of all trials as well as on the data of each trial separately. In all four cases the data points of the two groups overlap in the kernel space, as can be seen in Figure 14.

#### Spatial Distribution of Eye Movement Features in the Visual Field Used to Reconstruct the VFD

We could reconstruct the location of the VFD, if the VP was reduced in the damaged part of the visual field or if the frequency of fixations differed significantly from normal in those areas.

**Figure 15:**
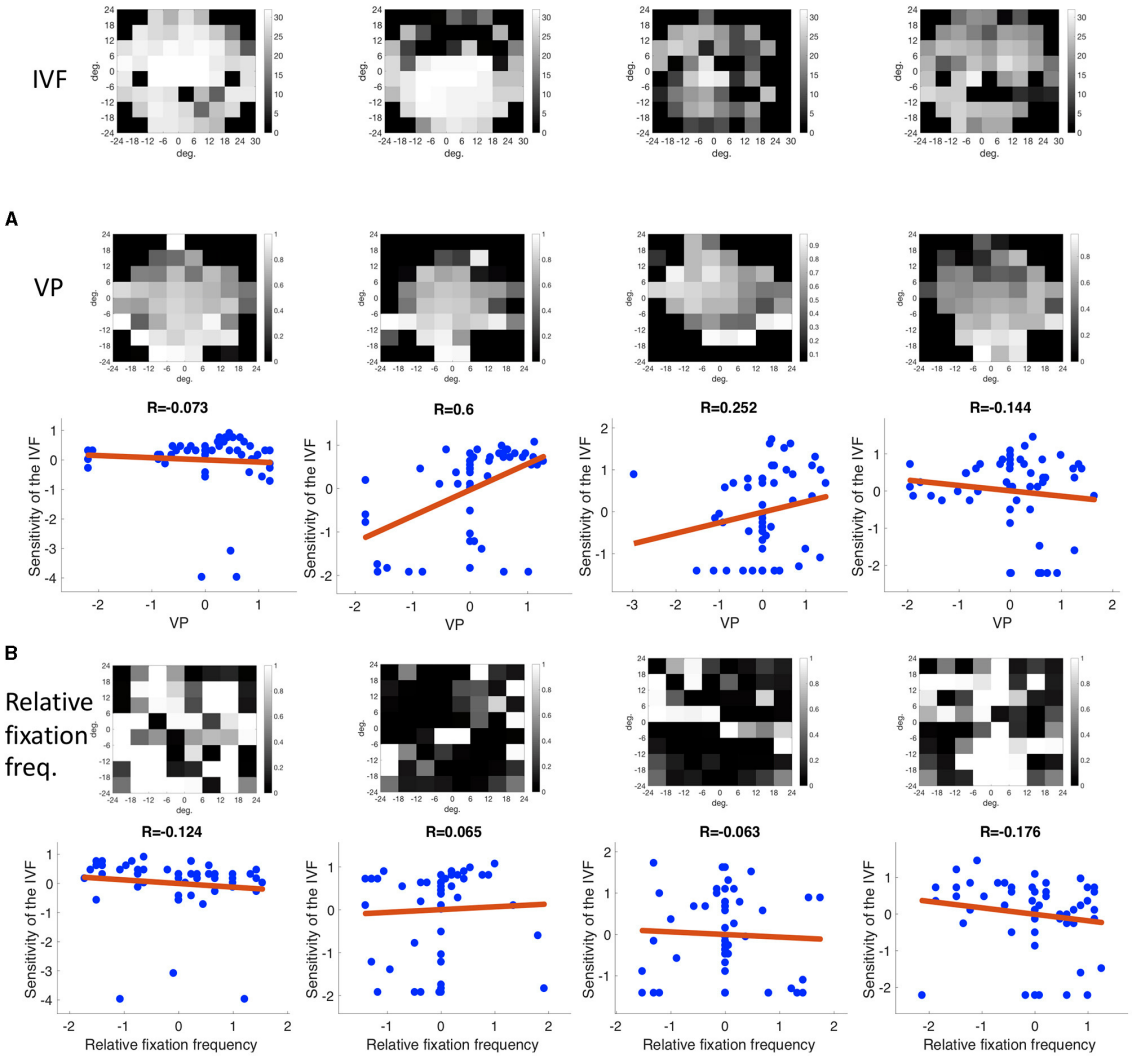
Representative examples of **(A)** the correlation of the VP and the sensitivity of the IVF and **(B)** the correlation of the relative fixation frequency and the sensitivity of the IVF. The discrete locations in which the VP and relative fixation frequency were binned matched the 24-2 SITA test locations of the HFA. Both panels show the same patients. Their IVF is shown in the top row. Neither the VP nor the relative fixation frequency is consistently correlated with the IVF over all patients.

Figure 15A shows examples of the distribution of VP and maps of patients with different severity and location of the VFD. As these representative examples show, there was a lot of variability between correlation coefficients of individual glaucoma patients. When taking the group average, the sensitivity per location of the IVF of the glaucoma patients was weakly correlated with the VP per location, with an average Pearson's correlation coefficient of *r* = 0.127. They however ranged between being weakly anti-correlated to being moderately correlated between individual patients (minimum: Pearson's *r* = −0.270, maximum: Pearson's *r* = 0.600).

Figure 15B shows the differential fixation maps of the same patients. Again, they represent the variability in correlation coefficients within the group. On the group level, the differential fixation maps were weakly anti-correlated with the sensitivity indifferent locations of the IVF, with an average correlation coefficient of Pearson's *r* = −0.040. They ranged from moderately anti-correlated to moderately correlated (minimum: Pearson's *r* = −0.502, maximum: Pearson's *r* = 0.410).

## Discussion

The main conclusion of this study is that monocular eye movements made by glaucoma patients differ substantially from those of normal-sighted controls. Based on this, we conclude that screening patients for VFD in glaucoma patients based on their free-viewing eye-movements is possible in principle.

The viewing conditions and the video clip content influence the ability to separate patients and controls. The differences in monocular viewing behavior between the two groups, becomes apparent in the significant difference in VP between the two groups, as well as the differences in directional saccade amplitudes between individual glaucoma patients and the control group. In addition, after transforming the data using kPCA, we found that participants with glaucoma and controls tend to form separate clusters in the new features space. The binocular viewing behavior, however did not lead to significant differences in the aforementioned features. Apart from the (binocular vs. monocular) viewing conditions, the second factor that influences if the two groups show differences in viewing behavior is the content of the video clips. Videos that contain highly dynamic content (such as comics) and that result in consistent viewing behavior in controls are the most suitable as these result in a good separation of the two groups. However, this success in detecting the presence of a VFD does not automatically translate into its reliable reconstruction. With any of the evaluated methods, reconstruction of the location of the VFD was only possible in a few patients, specifically in those with a large peripheral VFD and in the single patient with HH that we assessed. We will discuss these results below in more detail, including how compensatory viewing strategies of glaucoma patients may have prevented us from localizing their VFD.

### Detecting a VFD Requires Monocular Viewing

We analyzed the viewing behavior of glaucoma patients and controls in two comparable experiments with rather distinct, yet informative, outcomes. In experiment 1, we found significant differences in average saccade amplitude between patients and controls, a significant difference in VP after collecting data during 1-min video clips in 27 of the 28 video clips, differences in directional saccade amplitudes of the patients from the controls and potentially separable clusters after performing kPCA on the fixation maps. In experiment 2 we only found a significant difference in VP for one of the three video clips, which lasted 5:15 min and differences in directional saccade amplitudes compared to controls.

Why did we only find differences in eye movement behavior between patients and controls in experiment 1, where our participants viewed movies monocularly and not in experiment 2 where participants viewed these binocularly? To answer this question, we will first consider that due to glaucoma being a gradually progressing disease, many glaucoma patients do not notice their VFD until at a late stage during the disease. Moreover, they likely have adapted their viewing behavior to their VFD. In glaucoma, the VFD is caused by damage to the optic nerve. This results in defects at different locations and of different degrees of severity which may also be different for the two eyes. This was also the case for the glaucoma patients who participated in our present experiments. In experiment 1, this meant that from the moment we covered one eye, the state of a patient's visual field changed abruptly. Presumably, in the relatively brief time of the experiment, patients did not have sufficient time to adapt to this change. This means that during the experiment, their viewing experience differed from that in their daily life, analogous to what participants with simulated VFD experience. Indeed, after the experiment, some participants spontaneously mentioned that they had more difficulties watching the video clips monocularly, in comparison to watching television at home with both eyes.

We also found that patients who viewed the video clips with their worse eye usually showed a lower VP than those who performed the experiment with their better eye. This could be an indicator that if patients experience an instantaneous deterioration of the visual field, they become more aware of the presence of the VFD, and it reduces their ability to perform their customary viewing behavior. These findings are in line with previous studies that found differences in monocular viewing behavior of patients and controls. For example, a study where glaucoma patients viewed static images with one eye also found differences in the patterns of saccade amplitudes in different directions in patients compared to control, similar to our own findings (9).

Asfaw et al. (24, 25) showed that the same patient displays differences in viewing behavior depending on with which eye they performed a free viewing task. The spread of fixation locations was smaller in the worse eye of glaucoma patients compared to the better eye (24). This supports our finding that patients who viewed movies with their worse eye showed a less normal viewing behavior (as indicated by a lower VP) than patients who viewed the movies with their better eye.

Comparing the results of our two experiments, we conclude that glaucoma patients with asymmetric visual field loss, only show different eye movement behavior compared to normal-sighted controls, when they watch videos under viewing conditions that differ from the ones they experience in their daily life. When allowed to use both eyes, as in daily life, they seem to be able to view the videos as well as normal-sighted participants. Moreover, the presence of a VFD can be detected more easily in the worse eye. In patients with symmetric visual field loss, it may be irrelevant for their viewing behavior whether they view the video clips monocularly or binocularly.

### Detecting a VFD Under Free-Viewing Conditions Requires Suitable Video Content

An uncertainty concerning the findings of experiment 2, is whether we could have found differences in viewing behavior between the two groups if participants had watched the clips with one eye. It could also be the case that the content of the video clips did not lead to a good separation of the two groups. An indication of the videos shown not being suitable to separate the two groups, could be the low average VP of the controls in experiment 2 in comparison to the VP of the controls in experiment 1, indicating more inconsistent viewing behavior of normal-sighted observers when watching the three video clips of experiment 2.

We found that ideal video clips should guide the attention of the (normal-sighted) observer, so that their viewing behavior is very consistent providing a clear picture of where the salient region in a scene should be located. In addition, the salient region should change location on the screen quickly to trigger long saccades, as this is the kind of viewing behavior that is difficult to perform for glaucoma patients. Previous studies have shown that other types of experimental paradigms, besides free-viewing, that are successful at separating glaucoma patients from controls, also when they are tested binocularly, are those that require the participants to perform saccades. Najjar et al. (26) reported that glaucoma patients showed a reduced saccade velocity and amplitude in pro-saccade tasks, compared to the controls. The reduced amplitude means that the saccades were hypometric on average. In addition, glaucoma patients made more anti-saccade errors than controls. In a tracking test, where different patients were asked to follow a dot with their gaze, Soans et al. (27) were able to differentiate glaucoma patients from controls and patients with other types of visual field defects, due to their inability to follow the dot when it made a sudden jump toward the periphery of the visual field, i.e., when a saccade was required. They were, however, able to follow the dot, when it moved around slowly, i.e., when smooth pursuit eye movements were required.

In our free-viewing paradigm, it is difficult to trigger a sufficient number of long saccades, as we do not want to specifically instruct participants to, for example, also look toward the corners of the screen. When watching a video, the implicit task is of course to follow the storyline, which leads to quite consistent eye movements, as we showed in this study. We could select an appropriate video by trying to determine if the center of gaze of our observers would shift often and rapidly, based on the bottom-up features. We know that motion has a stronger effect on eye movements, than other features, such as color, orientation or intensity, but the sum of all features is the best predictor for gaze location (28). However, gaze behavior cannot exclusively be explained by bottom-up features, as it is also guided by cognitive goals (29). Furthermore, eye movements will not necessarily be directed toward the most salient location, but toward the location from which the brain can extract the most relevant information (30).

In particular in the case of video clips, gaze will also be influenced by the semantics of the scenes, the presence of characters, and the storyline. In movies with a very clear story line, it will be relatively easy to predict where to look next.

That is also a reason to resort to VP rather than saliency measures. VP expresses a combination of salience and relevance, taking both image features and semantics, into account (16). Practically, we can use VP to test which types of video clips lead to a good separation between the two groups. More specifically, we can select videos in which the controls have a high VP while that of the patients is significantly lower. In experiment 1, we found that comics or feature films that contain a lot of movement and active scenes usually result in rather distinct scan paths in patients and controls. On the other hand, the nature documentaries that we used contain mostly scenes or landscapes having relatively low color contrasts and depicting slowly moving herds of animals. Consequently, slow motion is spread out over the entire scene. These types of movies neither contain a spatially narrow and salient area of interest, an obvious story line with a main character that should be followed. This results in much more inconsistent viewing behavior of the control group, that results in a lower group average VP with a larger variance. In turn, this inconsistency in the viewing behavior of the controls makes it more difficult to detect deviations in the eye movement behavior of patients.

### Usability of Current Eye-Tracking Technology in Elderly and Clinical Populations

In experiment 1 we were unable to acquire good quality eye movement data in approximately one third of the patients with VFD and the controls. If eye tracking technology is to be used in (clinical) practice with an elderly demographic, the technology needs to be adapted accordingly.

For many participants, it took us a long time to modify the setup, adjusting the position of the eyes in the camera image of the tracker, trying to minimize reflections from the participants' glasses to be able to start the calibration. With most participants we had to perform the 9-point calibration several times, adjusting the setup in between. As the eye trackers that we used did not work with multifocal glasses, we used the trial lenses that are used to test patients' optimal correction at eye clinics. With all the necessary adjustments, in some participants it could take up to 25 min until we could collect data. With the goal in mind of using eye-tracking in clinical practice, either as a diagnostic tool or as support during vision rehabilitation, the time it takes to prepare for data collection needs to be reduced drastically. This could be achieved by using an eye tracker which does not need to be calibrated for every participant separately, provided these trackers manage to maintain stable gaze tracking {e.g., the stereo-eyetracker described in (31); the Pupil Invisible [Pupil Labs; (32)], or the BulbiCam (Bulbitech, Trondheim)}.

### Using Virtual Reality and Mobile Eye Tracking During Daily Life Activities Instead of a (Small) Screen Could Improve the Separability of Patients and Controls

A particular challenge, also with established methods, is to detect glaucoma at an early stage, as it usually starts by affecting the periphery. In both parts of this study, all participants showed a strong center bias, which meant that the periphery of the visual field was extremely undersampled. This problem may be reduced by selecting content in which the salient stimuli also occur around the edges of the screen and have a sudden onset. However, a remaining complication is that observers show a tendency to look toward the center of a screen irrespective of the presented content (33). Even when shifting image features away from it, people still tend to look toward the center of the screen (34). Therefore, one may question whether a regular computer display is the best device for presenting stimuli with the purpose of detecting a VFD. With advances in virtual reality (VR) technology that allow combining it with eye-tracking, presenting stimuli using a VR headset with a larger field of view and less obvious borders could be a more suitable alternative. This kind of display would potentially lead to even more natural viewing behavior and larger saccade amplitudes. Imaoka et al. (35) found that the HTC Vive Pro Eye VR headset could potentially be used to measure saccadic eye movements.

Another interesting approach could be to record eye movements during daily life activities with a mobile eye tracker. Having data of daily life eye movements could also help to reveal the degree to which a certain VFD diminishes functional vision in different tasks. In turn, his knowledge could be applied in vision rehabilitation therapy.

### VP May Correlate More Strongly With Functional Vision Than the Severity of the VFD Defined by the MD Value

While directly linking eye movement behavior to the patients' difficulties in daily life functioning lies outside the scope of this study, we can look for indicators of compensatory eye movement behavior. There are two explanations for why it was possible to detect the presence of a VFD based on eye movements. First, patients with VFD may have altered their eye movement behavior, due to the fact that they did not detect certain stimuli or interesting objects in the periphery. Consequently, they may have directed their overt attention less frequently toward damaged parts of their visual field. In other words, the viewing behavior of the patients would mainly have been altered due to bottom-up influences. Secondly, patients with VFD may employ viewing strategies with which they compensate for their VFD by directing their gaze more frequently toward certain (damaged) parts of the visual field. This strategy may either be used in a conscious or unconscious manner. If so, this would imply that their eye movement behavior would mainly have been altered by top-down mechanisms.

Importantly, if eye movements would be driven primarily by bottom-up influences, we would have expected a strong correlation between the severity of the VFD (MD or IVF score) and the VP value. In addition, when examining the distribution of fixations across the visual field, we had expected to find that patients fixate damaged parts of the VF much less frequently. However, we find that this is only the case in patients with severe peripheral VFD. Other patients frequently look toward damaged parts of their visual field, which could either be the effect of a compensatory strategy or reflect that filling-in hides the damaged part. Interestingly, patients with similar VFD showed markedly different average VP, also depending on whether they viewed the video clips with their better or worse eye. We speculate that VP could be a predictor for the ability of individuals to cope with their VFD in daily life. Given a similar VFD, the patient with a higher VP would be predicted to have a better compensatory eye movement strategy and better functional vision. When glaucoma patients watched the videos binocularly, they exhibited a very similar viewing behavior as the normal-sighted controls, not only showing a similar VP, but also similar saccade amplitudes and fixation distributions across the visual field. This suggests that for the task of binocularly watching videos, they may have already found mechanisms to cope with their VFD.

### Future Studies

Future studies could use the knowledge gained in this study to optimize the presented stimuli, as well as the stimulus display. With a larger data set it would also be possible to apply machine learning to predict the state of the visual field based on eye movement data. Based on the results of this study, the input features for a machine learning classifier should represent the spatial distribution of eye movements across the visual field.

Besides using eye movements to screen for the presence of a VFD, these could also be used to monitor the effects of vision rehabilitation training. One could test if free-viewing eye movement behavior changes over the course of different stages of vision rehabilitation. If it changed in a systematic fashion, we could conclude that the patient internalizes a certain compensatory eye movement strategy. In fact, we think it is appropriate to conclude that the degree to which a patient with a VFD could use and profit from a compensatory strategy in this experiment, depends on the size and the location of their VFD and the semantics and content of a specific video clip. This implies a potential trade-off to be made between precisely targeted, yet boring stimuli (such as a Gaussian blob), or more engaging but somewhat less accurate natural stimuli such as movies. Moreover, note that the above further implies that movies could be used to determine deviant viewing behavior (which is also evident from our saccade analyses) and potentially be used to quantify compensatory viewing behavior.

In addition, eye movement behavior during movie viewing could be used to predict how well patients can perform different daily life tasks. To answer this question, the quality of life and task performance in different daily life tasks should be assessed together with the eye movements.

### Conclusion

A VFD results in specific viewing behavior during video viewing that can be used to distinguish a glaucoma patient from control observers and which could form the basis of a simple screening approach. Distinguishing requires monocular viewing and considering the spatial distribution of eye movement features, such as fixation locations and saccade amplitudes. Moreover, we conclude that while individual glaucoma patients not only have different VFD, they also appear to differ in their ability to cope with it.

## Data Availability Statement

The raw data supporting the conclusions of this article will be made available by the authors, without undue reservation. The data and analysis scripts can be found on dataverse.nl.

## Ethics Statement

The studies involving human participants were reviewed and approved by Ethics Committee of the University Medical Center Groningen. The patients/participants provided their written informed consent to participate in this study.

## Author Contributions

BG acquired and analyzed the data for the work and contributed in writing the manuscript. J-BM substantially contribution to the conception or design of the work, the analysis and interpretation of data, and provide approval for publication of the content. FC substantially contribution to the conception of the work, contributed in writing the manuscript, and approved the submitted version. All authors contributed to the article and approved the submitted version.

## Funding

This project has received funding from the European Union's Horizon 2020 research and innovation program under the Marie Sklodowska-Curie grant agreement No. 661883 (EGRET) and the Graduate School of Medical Sciences (GSMS), of the University Medical Center Groningen, University of Groningen. The funding organizations had no role in the design, conduct, analysis, or publication of this research.

## Conflict of Interest

The authors declare that the research was conducted in the absence of any commercial or financial relationships that could be construed as a potential conflict of interest. The handling editor declared a past co-authorship with one of the authors FC.

## Publisher's Note

All claims expressed in this article are solely those of the authors and do not necessarily represent those of their affiliated organizations, or those of the publisher, the editors and the reviewers. Any product that may be evaluated in this article, or claim that may be made by its manufacturer, is not guaranteed or endorsed by the publisher.
