# Supplementary material for: How Free-Viewing Eye Movements Can Be Used to Detect the Presence of Visual Field Defects in Glaucoma Patients
Source: Front Med (Lausanne). 2021 Oct 21;8:689910. doi: 10.3389/fmed.2021.689910 (PMC8566763; doi:10.3389/fmed.2021.689910)
Supplement: Supplementary file 1 [file Table_1.docx]

**Supplementary Material**

*Table S1*

*List of movies*

Haai Five

Beestenboot

Jets

Buurman en Buurman j

Knabbel en Babbel

Donald Duck

Bambi

Beauty and the Beast

Jungle Book

Russen

Spangen

Baantjer

Dik Trom

Dansen op de vulkaan

5 kinderen en it

Freek in het wild

Freek op safari

Nieuwe wildernis
